# Genome-Wide RNAi Screen in IFN-γ-Treated Human Macrophages Identifies Genes Mediating Resistance to the Intracellular Pathogen *Francisella tularensis*

**DOI:** 10.1371/journal.pone.0031752

**Published:** 2012-02-16

**Authors:** Hongwei Zhou, Glen DeLoid, Erica Browning, David J. Gregory, Fengxiao Tan, Alice S. Bedugnis, Amy Imrich, Henry Koziel, Igor Kramnik, Quan Lu, Lester Kobzik

**Affiliations:** 1 Department of Environmental Health, Harvard School of Public Health, Boston, Massachusetts, United States of America; 2 Department of Medicine, Beth Israel Deaconess Medical Center, Harvard Medical School, Boston, Massachusetts, United States of America; 3 Department of Medicine, Boston University School of Medicine, Boston, Massachusetts, United States of America; 4 Department of Pathology, Brigham and Women's Hospital, Harvard Medical School, Boston, Massachusetts, United States of America; Institut de Pharmacologie et de Biologie Structurale, France

## Abstract

Interferon-gamma (IFN-γ) inhibits intracellular replication of *Francisella tularensis* in human monocyte-derived macrophages (HMDM) and in mice, but the mechanisms of this protective effect are poorly characterized. We used genome-wide RNA interference (RNAi) screening in the human macrophage cell line THP-1 to identify genes that mediate the beneficial effects of IFN-γ on *F. tularensis* infection. A primary screen identified ∼200 replicated candidate genes. These were prioritized according to mRNA expression in IFN-γ-primed and *F. tularensis*-challenged macrophages. A panel of 20 top hits was further assessed by re-testing using individual shRNAs or siRNAs in THP-1 cells, HMDMs and primary human lung macrophages. Six of eight validated genes tested were also found to confer resistance to *Listeria monocytogenes* infection, suggesting a broadly shared host gene program for intracellular pathogens. The *F. tularensis*-validated hits included ‘druggable’ targets such as TNFRSF9, which encodes CD137. Treating HMDM with a blocking antibody to CD137 confirmed a beneficial role of CD137 in macrophage clearance of *F. tularensis*. These studies reveal a number of important mediators of IFN-γ activated host defense against intracellular pathogens, and implicate CD137 as a potential therapeutic target and regulator of macrophage interactions with *Francisella tularensis*.

## Introduction

*Francisella tularensis* is a highly virulent, facultative intracellular bacterium that causes tularemia [1], [2]. *F. tularensis* is considered a potential bioweapon, in part because it is extraordinarily infectious, requiring inoculation or inhalation of as few as 10 bacteria to cause disease. Historical details of episodic outbreaks, possible military applications and bioterrorism implications are reviewed in [3]. Given the limitations of current vaccines and therapies, novel diagnostic and treatment modalities are needed, but progress has been stymied by a lack of knowledge about key pathogenic and host defense mechanisms.

The natural history of infections caused by *F. tularensis* offers a promising clue. Macrophages are the main site of replication of *F. tularensis* [4], and lung macrophages are especially targeted in inhalational tularemia [5]. *F. tularensis* infection of macrophages has been studied *in vitro*, where bacterial growth can produce more than 1,000 fold replication [6]. Intracellular killing of *F. tularensis* by macrophages depends on IFN-γ-induced activation [7], [8], [9], but the specific mechanisms of IFN-γ induced killing are not fully understood. Inducible nitric oxide synthase (iNOS) and NADPH phagocyte oxidase contribute to the antimicrobial activity of murine macrophages [10], [11], however, there is some controversy surrounding their importance [12], [13]. There is considerable discordance in different experimental systems, reflecting variable responses of macrophages from different species and sites [13], [14], [15] and the use of different conditions by different laboratories. Furthermore, there are important differences between human and rodent responses to *F. tularensis*, including macrophage cytokine responses [15] and comparative mortality *versus* immunization seen with the partially attenuated *F. tularensis* live vaccine strain (LVS) [14]. More recent data implicates autophagy [16], [17] and inflammasomes [18], [19], [20], although the importance of these in the specific context of IFN-γ stimulation has not been demonstrated. Human macrophages show potent antibacterial function once “activated”, but the genes responsible for this desirable trait are unknown. We hypothesized that IFN-γ limits intracellular pathogen *F. tularensis* by activation of specific genes, and that functional genomic screening could identify these genes.

The human macrophage cell line THP-1 provides a useful model for studies of *F. tularensis* intracellular pathogenesis and reproduces the key response of interest, activation by IFN-γ of bacteriostatic/killing mechanisms for intracellular pathogens [7], [8], [9]. To identify these IFN-γ- induced genes involved in resistance to intracellular *F. tularensis*, we performed a genome-wide RNA interference screen in THP-1 cells. THP-1 cells stably transduced and expressing shRNAs were phorbol-12-myristate acetate (PMA) differentiated, activated by IFN-γ, and infected with *F. tularensis* live vaccine strain (LVS) expressing green fluorescent protein (GFP) [21]. Infected THP-1 macrophages were sorted by green fluorescence and the top 1% isolated to enrich for cells in which shRNA knockdown blocked the ability of IFN-γ-induced genes to inhibit GFP-LVS proliferation. From this screen, 212 candidate genes and ESTs were identified, of which 168 were selected for expression analysis by real-time PCR array, both before and after infection, in three different models of IFN-γ activated human macrophages: THP-1 cells, human monocyte-derived macrophages, and primary human alveolar macrophages,. A panel of 20 genes (top hits of interest) was further subjected to functional validation by specific siRNA or lentivirus-mediated shRNA knockdown. Our results identified several ‘druggable’ genes as potential therapeutic targets, and new leads to host defense mechanisms. We further tested one top “hit,” the receptor CD137 (TNFRSF9) in detail, using a blocking anti-CD137 receptor antibody, and confirmed its function in human macrophage clearance of *F. tularensis*.

## Results

### Interferon-γ activated THP-1 macrophages inhibit intracellular replication of *F. tularensis*

**Figure 1:**
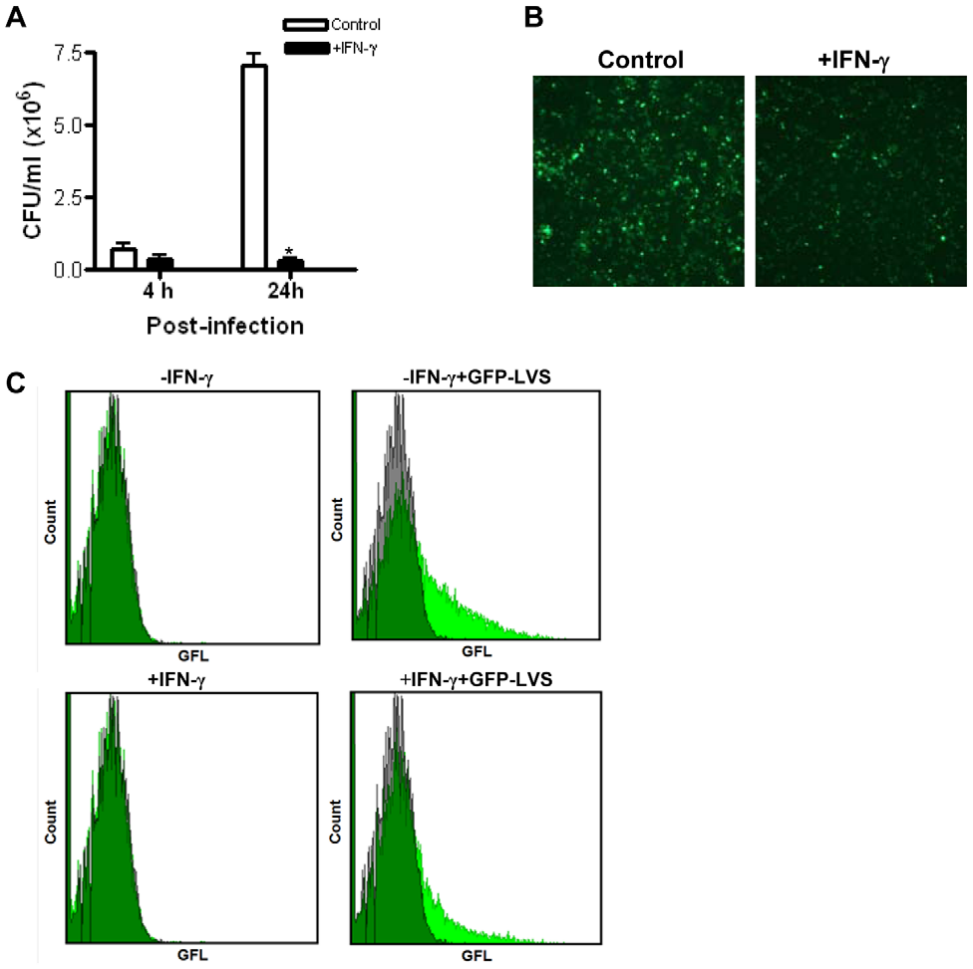
IFN-γ-activated THP-1 cells inhibit intracellular replication of *F. tularensis*. Quiescent (control) and IFN-γ-activated (100 U/ml) THP-1 cells were incubated for 2 h with GFP-expressing *F. tularensis* LVS, followed by 30 min gentamicin treatment to kill extracellular bacteria, and subsequent determination of the number of intracellular bacteria at 4 h and 24 h post-infection by (A) colony forming unit (CFU) assay. Multiplicity of infection (MOI) was 10∶1 (bacteria: macrophage). *P<0.05 compared to control. Data were pooled from three independent experiments and presented as mean±SD. (B) Representative images showing the number of intracellular GFP-LVS after 24 h infection by fluorescence microscopy (400×). MOI = 40∶1. (C) Quantification of the intracellular GFP-LVS bacteria after 24 h infection (MOI = 40∶1) by flow cytometry, with axes set to the same scales for all groups and10,000 cells counted per sample. Gray histograms represent uninfected cells. Green histograms represent cells infected with GFP-LVS, and overlap between the two in dark green.

We first tested our assumption that IFN-γ activation would enable the human macrophage cell line THP-1 to inhibit intracellular replication of *F. tularensis*. THP-1 cells were differentiated by PMA, followed by pre-treatment with IFN-γ (100 U/ml) for 24 h, and the cells were infected with green fluorescent protein labeled *F. tularensis* LVS (GFP-LVS) as described in Methods. After 4 h and 24 h infection, the growth of GFP-LVS was examined by colony forming unit (CFU) assay (Fig. 1A). At 4 h after infection the number of intracellular bacteria in IFN-γ-activated THP-1decreased relative to control THP-1 cells. At 24 h after infection, the survival of *F. tularensis* in IFN-γ-activated THP-1 cells was approximately 20 times lower than observed in the unactivated (control) THP-1 cells at low MOI of 10 bacteria per macrophage. CFU assay indicated that bacterial replication was completely inhibited in IFN-γ-activated THP-1 macrophages. This result was confirmed by fluorescence microscopy and flow cytometry at 24 h post-infection (Fig. 1B and Fig. 1C), in which the green fluorescence of intracellular bacteria was markedly lower in IFN-γ treated THP-1 cells compared to control cells. The effect of IFN-γ is concentration dependent over the dose range of 20–500 U/ml (data not shown). These results verified the expected beneficial IFN-γ effect and its ability to limit *F. tularensis* LVS growth in the PMA-differentiated THP-1 macrophages.

### Preparation of human macrophage cell line THP-1 pool with genome-wide lentiviral shRNA library

In order to optimize transduction of the GeneNet™ lentiviral shRNA library into THP-1 cells we evaluated transduction efficiency of control viral particles over a range of virus titers using the two available expression systems (FIV- and HIV-based) from System Biosciences (SBI). THP-1 cells were transduced with copGFP by positive control lentiviral particles and transduction efficiency was assessed by fluorescence microscopy (Fig. S1A and S1B) and flow cytometry (Fig. S1C) 4 days post-transduction. Percent transduction increased with multiplicity of infection (MOI), but MOIs of 20 or greater resulted in substantial cell death. At virus titers of 10–15 per macrophage, transduction efficiency was greater with the FIV-based (80%–86%) than with the HIV-based (52%–71%) expression system (Fig. S1C), and caused minimal cell death. Based on these results we selected the FIV-based vector and a viral titer of 10 for subsequent screen transductions. The average number of lentiviral constructs integrated into the genomic DNA of each transduced cell was estimated using the SBI Lentivector Rapid Titer PCR Kit. By comparing the quantity of vector-specific WPRE amplified from a sample of our transduced cells with that from the SBI calibration standard (Fig. S1D) we estimated an MOI for our screen transduction of ∼1.3, which, based on control data provided by SBI (Fig. S1D, lower panel), corresponded to a transduction efficiency of ∼75%, approximating the value predicted from the copGFP transduction data (Fig. S1C).

### Flow cytometric sorting to screen for genes that mediate the effect of IFN-γ on macrophage killing of *F. tularensis*

**Figure 2:**
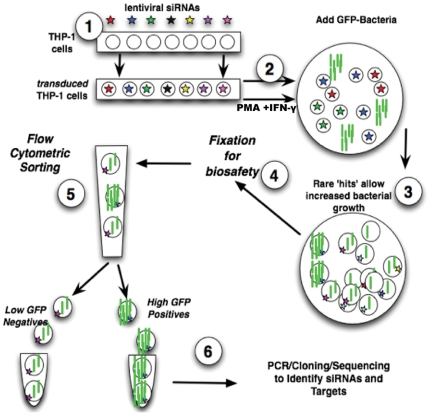
Protocol used for genome-wide RNAi screen: sorting for high GFP-positives. Step 1: construct THP-1 library cells by transduction of human THP-1 macrophage cell line with GeneNet™ Human 50 k shRNA library. Step 2: differentiate THP-1 library cells with PMA, and activate the cells with IFN-γ. Step 3: infect the library cells with GFP-labeled *F. tularensis* live vaccine strain (GFP-LVS). Step 4: harvest cells at indicated time points, and fix the cells for biosafety prior to flow cytometry. Step 5: FACS sorting to isolate small population of cells with the highest green fluorescence (containing the most GFP-LVS). Step 6: identify siRNA targets and genes by PCR, cloning and DNA sequencing.

Our strategy and protocol for the genome-wide screen is outlined in Figure 2. Following transduction of THP-1 cells with human 50K shRNA lentiviral library, transduced THP-1 cells were differentiated with PMA and pre-treated with IFN-γ, then infected with green fluorescent *F. tularensis* LVS for 2 h. Cells were then washed and treated with gentamicin to remove or kill extracellular bacteria, and incubated for 24 hours. Cells transduced with an shRNA that knocked down expression of a gene involved in the beneficial bactericidal effect of IFN-γ would be less able to prevent bacterial growth, so carry a higher load of *F. tularensis*, and thus exhibit higher green fluorescence. These cells were collected by FACS and their genomic DNA isolated. Target shRNA sequences were amplified from the lentiviral constructs integrated into the genomic DNA by PCR, and subcloned and expanded in *E. coli* to produce samples for sequencing, as detailed in materials and methods.

The results of five independent screens and FACS sorts were analyzed. Frequency analysis was used to identify genes of interest. We focused on the genes that appeared multiple times within an experiment, and placed the greatest emphasis on genes that appeared in multiple independent experiments, reasoning that biologically significant ‘hits’ would appear repeatedly and multiply, whereas single hits would likely be false positives resulting from noise within the system. Using this rationale, we identified top hits for further validation based on the following results. A total of 4078 sequences (740, 756, 960, 764 and 858, respectively) were obtained from the five independent sorts. From these, 3386 genes and ESTs were identified. Repeat hits (appearing in more than one screen) were found in the following frequencies: ≥2 sorts: 212 hits; ≥3 sorts: 61 hits; 4 sorts: 2 hits. In addition to hits identified in two or more screens, we included in our genes of interest 35 single hits, corresponding to genes that were very similar, albeit non-identical (but usually closely related), to genes that did appear in two or more screens. The rationale for including these genes is that if knockdown of functionally similar genes, differing slightly in minor sequence components, is observed repeatedly, then those genes or their products may belong to a functional group that constitutes a potentially valid target. The final list of 247 genes of interest is shown in Table S1.

**Figure 3:**
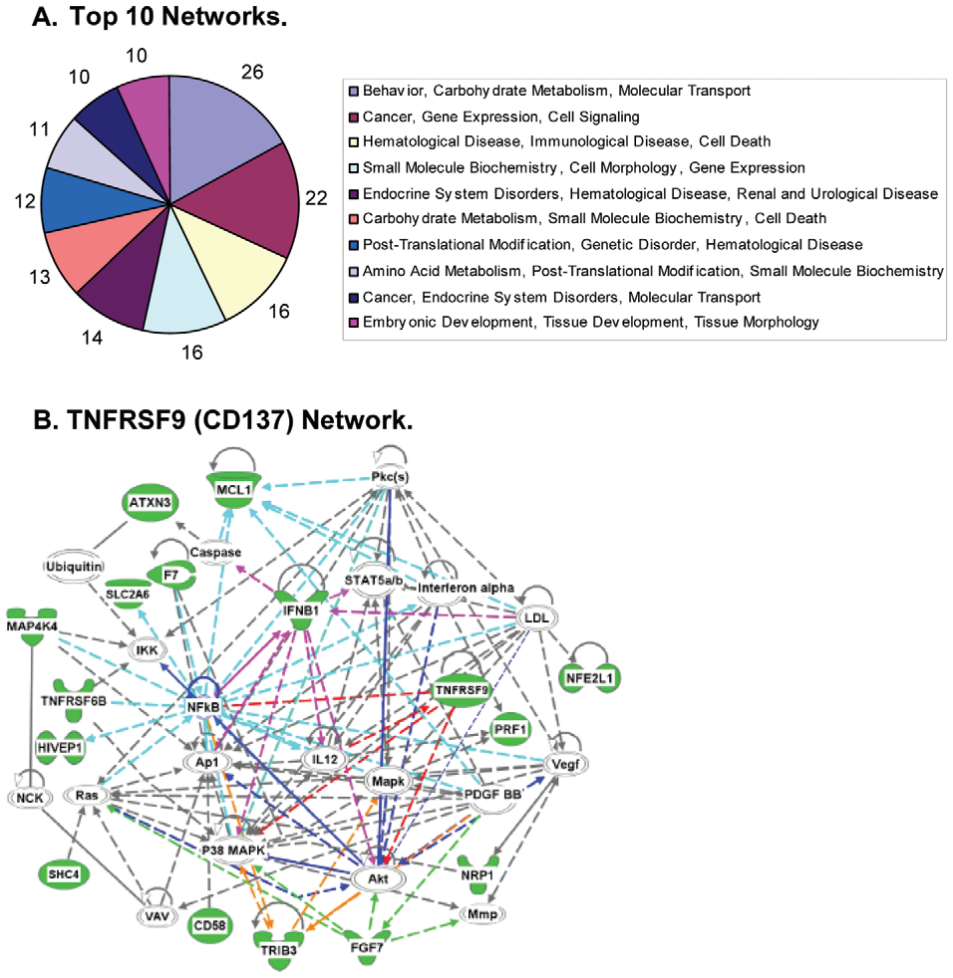
Network Analysis. (A) Top hits involved in ten significant networks identified by Ingenuity pathway analysis. The numbers surrounding the pie charts represent the number of the top hits involved in each network. (B) 16 hits (highlighted in green) are in a network linked to a category designated as ‘hematological disease, immunological disease, cell death’, which includes TNFRSF9/CD137. Connection lines ⁃⁃, represent direct interactions; ----, represent indirect interactions.

To further analyze the top 212 human genes found in 2 or more independent screens in our primary RNAi screen that potentially affect *F. tularensis* proliferation, these genes were uploaded into the Ingenuity Pathway Analysis (IPA) software (http://www.ingenuity.com), and canonical pathways were determined as described previously [22], [23], [24]. This analysis identified 110 hits with Molecular and Cellular Functions (Fig. S2A), 135 hits involved in Diseases and Disorders (Fig. S2B), and 52 hits associated with Physiological System Development and Function (Fig. S2C). In addition, 150 of the identified genes were found to be involved in ten networks (Fig. 3A), suggesting functional linkages among clusters of genes identified by our screen. There are limitations to this approach, since many genes cross over into multiple categories. Nevertheless, oOne of these networks includes 16 molecules in the category ‘Hematological Disease, Immunological Disease, and Cell Death’ and includes TNFRSF9 which may be involved in NF-κB, Akt, P38 MAPK and IL12 signaling pathways (Fig. 3B). TNFRSF9 is also known as CD137 which is further discussed below.

### Validation of target gene expression in human lung macrophages by qPCR

**Figure 4:**
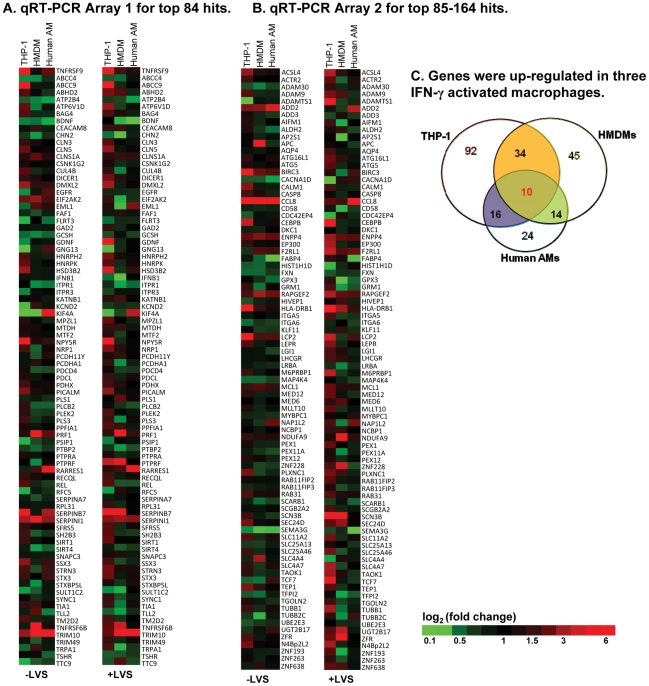
Gene expression validation of top 168 hits. Custom human qRT-PCR arrays (Array 1, top 84 hits;Array 2, top 85–164 genes) were used to measure gene expression in three human macrophages (THP-1 cell line, human monocyte-derived macrophages, and human primary AMs) with or without IFN-γ activation, and with or without *F. tularensis* LVS infection for 24 hours (MOI = 40∶1). All gene expression was normalized to three human housekeeping genes, ACTB, GAPDH and NONO. (A & B) Heat map showing IFN-γ–induced gene expression changes among top 84 (A) and 85–164 (B) hits in the three types of IFN-γ-activated macrophages ± LVS (left and right columns) compared to control cells without IFN-γ treatment. Green indicates lower, and red, higher expression. (**C**) Venn diagram of genes up-regulated in the three types of IFN-γ activated macrophages with and without *F. tularensis* challenge. Ten genes showed induced expression in all three macrophages.

We selected a subset of the top 168 gene hits, shown in Table 1, for further validation. The primary criterion for selection was frequency: all hits found in 4 and 3 sorts, and most of those found in 2 sorts were included. Also included were a small number of genes of interest identified in parallel efforts using our EST-based library (*e.g*. pleckstrin, unpublished data) that were in the same functional area (*e.g*., intracellular trafficking) as other hits in the shRNA library screens. The choice of 168 hits in part also reflects the number of genes that could be analyzed per plate in the custom array platform used (84 per plate, see Methods). Expression of putative gene hits was measured in macrophages to test the prediction that these genes would, at a minimum, be expressed, and would likely exhibit increased expression after IFN-γ pretreatment, and/or after IFN-γ pretreatment followed by *F. tularensis* challenge (consistent with the observed biological effect of their knockdown). Three types of macrophages were used in these experiments: PMA-differentiated THP-1 macrophages, human monocyte-derived macrophages, differentiated to be ‘alveolar-macrophage’-like [25], and primary human alveolar macrophages (AMs). The 168 gene expression levels in IFN-γ-induced macrophages were compared to those in control cells without IFN-γ activation. The results of quantitative PCR from these studies are summarized in Figure 4A and Figure 4B. In THP-1 macrophages basal or induced expression was seen for the majority of the original hits with IFN-γ activation (a fold change of ≥1.5 was set as the threshold for gene up-regulation). Numerous hits were also expressed differentially by HMDMs and primary human AMs. Ten genes were identified with overlapping expression changes in all three cell types. These are shown in Figure 4C and detailed in Table 2. These results confirm the importance of comparing results with the useful but imperfect cell line model (THP-1) to actual primary cells.

**Table 1:** Top 168 genes chosen for expression analysis by qRT-PCR arrays.

ABCC4	ENPP4	MYBPC1	SERPINB7
ABCC9	EP300	N4Bp2L2	SERPINI1
ABHD2	F2RL1	NAP1L2	SFRS5
ACSL4	FABP4	NCBP1	SH2B3
ACTR2	FAF1	NDUFA9	SIRT1
ADAM30	FLRT3	NPY5R	SIRT4
ADAM9	FXN	NRP1	SLC11A2
ADAMTS1	GAD2	PCDH11Y	SLC25A13
ADD2	GCSH	PCDHA1	SLC25A46
ADD3	GDNF	PDCD4	SLC4A4
AIFM1	GNG13	PDCL	SLC4A7
ALDH2	GPX3	PDHX	SNAPC3
AP2S1	GRM1	PEX1	SSX3
APC	HIST1H1D	PEX11A	STRN3
AQP4	HIVEP1	PEX12	STX3
ATG16L1	HLA-DRB1	PICALM	STXBP5L
ATG5	HNRPH2	PLCB2	SULT1C2
ATP2B4	HNRPK	PLEK2	SYNC1
ATP6V1D	HSD3B2	PLS1	TAOK1
BAG4	IFNB1	PLS3	TCF7
BDNF	ITGA5	PLXNC1	TEP1
BIRC3	ITGA6	PPFIA1	TFPI2
CACNA1D	ITPR1	PRF1	TGOLN2
CALM1	ITPR3	PSIP1	TIA1
CASP8	KATNB1	PTBP2	TLL2
CCL8	KCND2	PTPRA	TM2D2
CD58	KIF4A	PTPRF	TNFRSF6B
CDC42EP4	KLF11	RAB11FIP2	TNFRSF9
CEACAM8	LCP2	RAB11FIP3	TRIM10
CEBPB	LEPR	RAB31	TRIM49
CHN2	LGI1	RAPGEF2	TRPA1
CLN3	LHCGR	RARRES1	TSHR
CLN5	LRBA	RECQL	TTC9
CLNS1A	M6PRBP1	REL	TUBB1
CSNK1G2	MAP4K4	RFC5	TUBB2C
CUL4B	MCL1	RPL31	UBE2E3
DICER1	MED12	SCARB1	UGT2B17
DKC1	MED6	SCGB2A2	ZFR
DMXL2	MLLT10	SCN3B	ZNF193
EGFR	MPZL1	SEC24D	ZNF228
EIF2AK2	MTDH	SEMA3G	ZNF263
EML1	MTF2	SERPINA7	ZNF638

**Table 2:** Top 10 genes with increased expression in all 3 human macrophage types.

Gene	Identity	Function
**TNFRSF9**	NM_001561	Tumor necrosis factor receptor superfamily 9
**CLNS1A**	NM_001293	Chloride channel, nucleotide-sensitive, 1A
**RARRES1**	NM_206963	Retinoic acid receptor-responsive gene
**SERPINI1**	NM_005025	Serpin (serine or cysteine) proteinase inhibitor, clade I (neuroserpin) member 1
**TRIM10**	NM_052828	Tripartite motif-containing 10 isoform 1 and 2
**CCL8**	NM_005623	Small inducible cytokine A8 precursor
**ENPP4**	NM_014936	Ectonucleotide phyrophosphatase/phosphodiesterase 4 (putative function)
**F2RL1**	NM_005242	Coagulation factor II (thrombin) receptor-like 1
**RAPGEF2**	NM_014247	Rap guanine nucleotide exchange factor 2
**HLA-DRB1**	NM_002124	MHC class II HLA-DR beta 1 or 4 chain

### Functional validation of top 20 hits with lentiviral shRNA or siRNA knockdown

**Figure 5:**
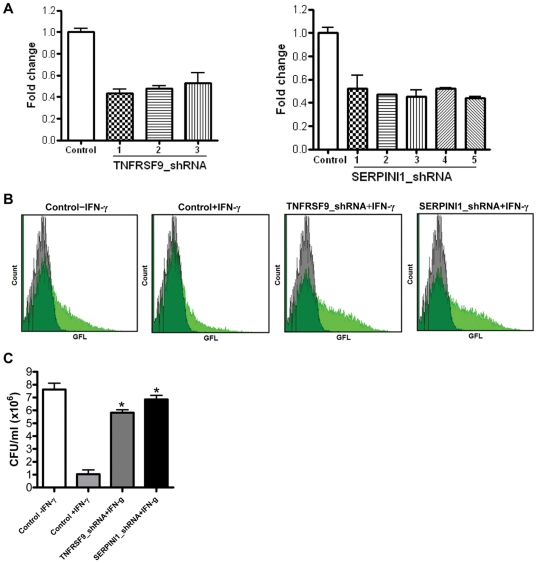
Validation of TNFRSF9 and SERPINI1 gene expression and function. (A) qPCR analysis of expression of TNFRSF9 and SERPINI1 genes in THP-1 macrophages after transduction with individual lentiviral shRNAs compared to controls transduced with non-targeting shRNA. PCR reactions were normalized against β-actin and plotted relative to expression levels in control cells. Error bars indicate± SD of triplicates. (B) Flow cytometry analysis of the effect of lentiviral shRNA knockdown of TNFRSF9 and SERPINI1 genes on *F. tularensis* GFP-LVS infection after 24 h in THP-1 cells ± IFN-γ, 40 U/ml. Controls were transduced with non-targeting shRNA. Quantification of GFP-LVS bacteria at 24 h post-infection (MOI = 82∶1) is shown by green fluorescence histograms, using identical scale axes for all groups; uninfected cell green fluorescence histograms are shown in gray, GFP-LVS infected cell histograms in light green, and overlap between the two in dark green. (**C**) CFU assay results of the effect of lentiviral shRNA knockdown of TNFRSF9 and SERPINI1 genes on GFP-LVS infection in THP-1 cells ± IFN-γ, 40 U/ml at 24 h (MOI = 82∶1). Control THP-1 cells were transduced with non-targeting shRNA. Mean ± SD, *P<0.05 compared to control+IFN-γ group.

Our next goal in validation was to test whether we could reproduce the functional phenotype selected originally by the screening assay. If these genes are truly important functionally, then individual knockdown should replicate the screen – namely, cause loss of antibacterial capacity and allow growth of intracellular *F. tularensis*. We used two complementary approaches. The first was to obtain commercially-available individual lentiviral shRNA preparations targeting the genes, transduce THP-1 cells, select transductants with puromycin and establish pools of lentiviral transduced cells. Using this approach, we first tested whether the lentiviral shRNA was having its expected effect of reducing gene expression. Figure 5A shows the results with 3 different sequences against one target, TNFRSF9, and in this case we observed satisfactory reduction of gene expression (50%–60%) with all 5 shRNAs tested. Similar gene knockdown was observed for SERPINI1 with 5 different lentiviral shRNAs. We have tested *F. tularensis* bacterial growth within subcloned individual knockdown lines from THP-1 cells transduced with these shRNAs. The flow cytometry data show that knockdown of the gene targets TNFRSF9 and SERPINI1 does have the predicted effect of increasing bacterial content, despite IFN-γ activation of the macrophages (Fig. 5B). In these experiments we initially used a green fluorescent *F. tularensis* and measured intracellular bacterial content after 24 h infection by flow cytometry. This type of knockdown validation has been successful for all 5 of the first set of top hits (TNFRSF9, CLNS1A, RARRES1, SERPINI1 and TRIM10). We have also been able to validate these findings further by bacterial CFU assays. The results of CFU assay for TNFRSF9 clone D and SERPINI1 clone 1 showed increased bacteria growth in THP-1 lines in which TNFRSF9 and SERPINI1 genes were knocked down by shRNAs (Fig. 5C).

**Figure 6:**
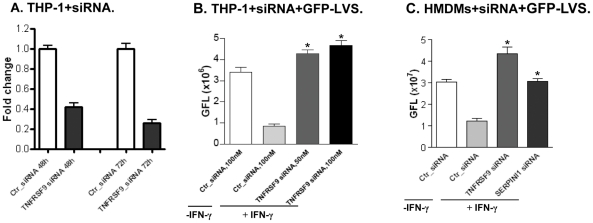
Validation of TNFRSF9 and SERPINI1 gene expression and function with gene knockdown by siRNA. (A) Comparison of TNFRSF9 gene expression using qPCR assay after treatment of THP-1 cells with 100 nM specific or control siRNA for 48 hours and 72 hours. (B) Flow cytometry analysis of siRNA TNFRSF9 knockdown in THP-1 cell with *F. tularensis* GFP-LVS infection for 24 h. THP-1 macrophages were treated with 50 nM or100 nM of siRNA for 72 h, and pre-activated with IFN-γ (40 U/ml) overnight prior to GFP-LVS infection (MOI = 35∶1). Y-axis represents mean green fluorescence level (GFL). *P<0.05 compared to control Ctr_siRNA+IFN-γ group. (C) Flow cytometry analysis of GFP-LVS infection for 24 h in HMDMs following 100 nM siRNA knockdown of TNFRSF9 and SERPINI1, respectively. *P<0.05 compared to control Ctr_siRNA+IFN-γ group.

Further functional evaluation of top hits was performed using liposome-based transfection of macrophages with individual siRNAs. This simpler delivery method allows validation testing in days, compared to the several weeks required to create stably transduced knockdowns, and also allows testing of non-replicating, terminally differentiated cells (*e.g.* HMDMs). Figure 6A illustrates one example of effective target (TNFRSF9) expression knockdown using this method. In this case expression of TNFRSF9 mRNA was reduced by ∼60% and ∼70% at 48 h and 72 h, respectively. Figure 6B demonstrates that THP-1 macrophages transfected with different concentration of siRNA (50 nM or 100 nM) targeting TNFRSF9 can reproduce the screening phenotype (reversal of the IFN-γ effect, with increased intracellular *F. tularensis* proliferation), confirming that TNFRSF9 acts to inhibit *F. tularensis* proliferation in activated macrophages. Because liposomal transfection can be performed in non-dividing cells, we were also able to validate hits in primary HMDMs. Figure 6C shows that HMDMs transfected with siRNAs targeting TNFRSF9 and SERPINI1 failed to control *F. tularensis* growth, despite IFN-γ treatment. The combined results of validation using lentiviral shRNAs and siRNAs in THP-1 cells and primary HMDMs are summarized in Table 3. The top 20 ‘hits’ shown in this table constitute a group of novel potential therapeutic targets. As shown in Table 4, existing modulating agents are already available for a subset of these targets, providing a direct means for investigating the potential benefit of targeting these genes or their products in *F. tularensis* infections.

**Table 3:** Summary of functional validation results for top candidates.

Gene Target	THP-1+lentiviral shRNA	THP-1+siRNA	HMDM+siRNA
TNFRSF9	++	++	++
SERPINI1	++	++	++
TRIM10	++	++	++
HLA-DRB1	++	++	++
PLEK2	++	++	++
PLS1	++	++	+
ATG5	++	++	++
ATG16L1	++	++	++
SERPINA7	++	++	+
SERPINB7	++	−	+
RAPGEF2	++	+	−
F2RL1	++	+	−
ENPP4	+	++	−
CCL8	++	+	−
CLN5	++	NT	NT
CLNS1A	NT	+	+
NAP1L2	−	++	+
RARRES1	NT	+	−
LCP2	NT	+	−
SLC4A7	NT	−	+

**Note**: Following shRNA or siRNA knockdown of the target genes, IFN-γ pretreated THP-1 and HMDM cells were infected with *F. tularensis* GFP-LVS. After 24 hours, the intracellular GFP-LVS was measured by flow cytometry and compared to control. Results are summarized as ++, GFP greater than control by ≥40%; +, GFP greater than control by ≥20%; −, GFP greater than control by <20%. NT, not tested.

**Table 4:** Top 5 hits as therapeutic leads.

Gene	Protein	‘Druggable’ Genome?	Therapeutics?
**TNFRSF9**	tumor necrosis factor receptor superfamily, member 9	YES	Agonist monoclonal (anti-CD137) in clinical trials [35]
**SERPINI1**	serpin peptidase inhibitor, clade I member 1	YES	Related serine protease family inhibitors used clinically (aprotinin, A1AT)
**RARRES1**	retinoic acid receptor responder	YES	Up-regulated by tazarotene [36], FDA-approved drug
**CLNS1A**	chloride channel, nucleotide- sensitive, 1A	YES	Multiple chloride channel enhancing drugs for CF [37]
**TRIM10**	tripartite motif-containing 10	YES	Zinc/metal binding domain suggests chelator function; chelators picolinic acid, desferal in clinical use

### Validation of top hits with virulent *F. tularensis* strain

**Figure 7:**
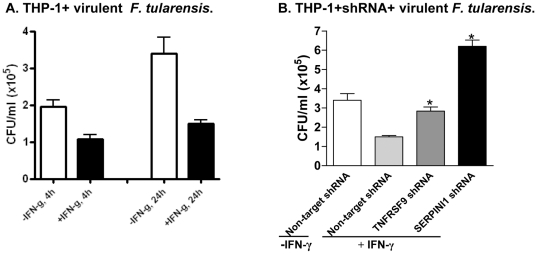
Validation of TNFRSF9 and SERPINI1 genes using virulent *F. tularensis*. (A) IFN-γ-activated THP-1 cells inhibited SchuS4 growth at 4 h and 24 h infection. IFN-γ, 40 U/ml (SchuS4, MOI = 30∶1). (B) CFU assay of TNFRSF9 and SERPINI1 shRNA knockdown THP-1 macrophages infected with SchuS4 strain. Control (THP-1 cells transduced with negative control lentiviral shRNA), TNFRSF9 knockdown cells (clone D) and SERPINI1 knockdown cells (clone1) were infected with virulent SchuS4 strain for 24 h (MOI = 30∶1). *P<0.05 compared to +IFN-γ group.

The regulatory and logistical challenges associated with using fully virulent, select agent strains of *F. tularensis* make use of such strains impractical for studies requiring extensive molecular biology. We therefore chose to perform the majority of this study using the attenuated model Live Vaccine Strain *F. tularensis* LVS, and to confirm key findings with fully a virulent strain, SchuS4. As expected, IFN-γ inhibited intracellular growth of SchuS4 in THP-1 cells, though not completely as LVS. After 24 h infection, IFN-γ-activated THP-1 cells had 60% lower virulent bacterial growth than quiescent THP-1 cells (Fig. 7A). Then we evaluated virulent *F. tularensis* SchuS4 infection of THP-1 cells in which six top hit genes (TNFRSF9, SERPINI1, HLA-DBR1, PLEK2, ATG5 and ATG16L1) were knocked down by individual lentiviral shRNA. At 24 h post-infection, knockdown of TNFRSF9 and SERPINI1 reversed the ability of IFN-γ to restrict survival of *F. tularensis* SchuS4 in THP-1 cells, as shown by CFU assay (Fig. 7B). Similar results were seen for the other genes tested, except PLEK2 (data not shown). These results suggest top hits identified using LVS are also relevant to virulent *F. tularensis* infection.

### Top hits mediate resistance to other intracellular pathogens

**Figure 8:**
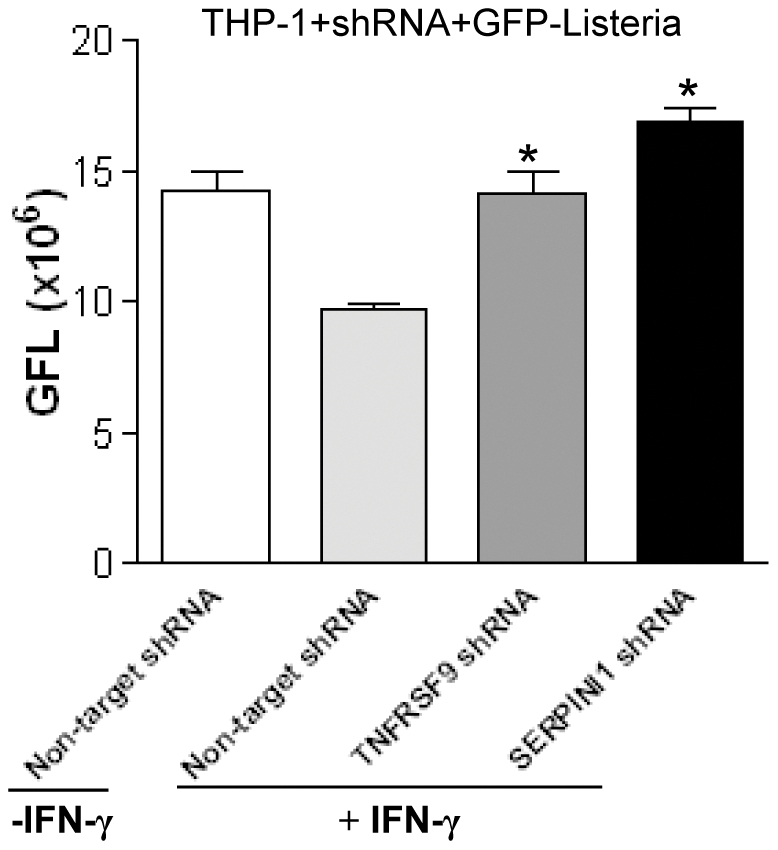
Effect of TNFRSF9 and SERPINI1 knockdown on Listeria growth in THP-1 macrophages. Flow cytometry analysis of THP-1 macrophages (transduced with TNFRSF9 and SERPINI1 shRNA or non-target control shRNA) 20 hours following infection with GFP-*Listeria* (MOI = 150∶1). Y-axis indicates mean green fluorescence index (GFL). *P<0.05 compared to Non-target shRNA+IFN-γ group.

To determine whether the IFN-γ benefit conferred or mediated by our top hit genes is specific to *F. tularensis* infection or functions more generally in response to intracellular pathogens, we investigated the effect of 8 of the top hit genes from our screen (TNFRSF9, SERPINI1, SERPINA7, HLA-DRB1, ATG5, ATG16L1, PLEK2 and PLS1) on *Listeria monocytogenes* infection in THP-1 cells stably transduced with shRNAs targeting these genes. Interestingly, at 20 h post-infection, six of the eight (all but PLEK2 and PLS1) also showed reversal of IFN-γ mediated resistance to *L. monocytogenes* infection, as illustrated in Figure 8 for THP-1 cells with TNFRSF9 and SERPINI1 gene knockdown.

### Novel role of CD137 in macrophage clearance of *F. tularensis*

**Figure 9:**
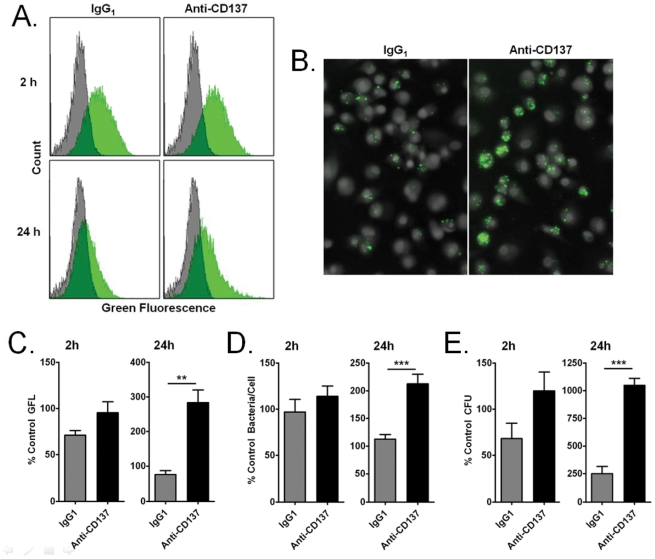
CD137 modulates clearance of *F. tularensis*. Adherent HMDMs were pre-treated with anti-CD137 antibody or isotype control prior to infection with *F. tularensis* GFP-LVS, and analyzed for bacterial load at 2 h and 24 h by flow cytometry, scanning cytometry and CFU assay. (A) Flow cytometry fluorescence histograms for a single experiment at 2 and 24 h. Gray histograms represent uninfected cells. Green histograms represent cells infected with GFP-LVS. (B) Representative scanning cytometry images of GFP-LVS-containing HMDMs at 24 h post infection. (C–E) Quantitative results at 2 h and 24 h for (C) flow cytometry (N = 4), (D) scanning cytometry (N = 5), and (E) CFU assay (N = 3). *P<0.05, **P<0.01, ** *P*<0.001 (symbols directly above bars indicate significance relative to untreated control; symbols between bars indicate significance between the two bars indicated by horizontal lines).

We sought to further explore the role of the gene identified in our screen as TNFRSF9, which encodes CD137. CD137 and its ligand, CD137L, are transmembrane modulatory proteins expressed on many immune cell types, and comprise a bi-directional signaling system, wherein CD137L initiates signaling through CD137, and vice versa [26], [27], [28]. We used a blocking monoclonal antibody to test the prediction that blocking this surface receptor would also reproduce the findings with our RNAi screen: namely, an increase in intracellular bacterial proliferation despite IFN-γ activation. As seen in Figure 9, both flow cytometry and scanning cytometry (9A–9D) show increased content of GFP-LVS in HMDMs treated with blocking anti-CD137 monoclonal antibody compared to IgG1 or no treatment controls. Measurement of viable LVS bacteria in cell lysates gave similar results (Fig. 9E). Our results demonstrate that CD137 can modulate clearance of *F. tularensis*, providing additional confirmation of this target initially implicated in the RNAi screen.

## Discussion

New therapies and countermeasures against biological warfare agents are needed, but progress has been stymied by a lack of knowledge about key pathogenic mechanisms. The natural history of infections caused by intracellular bacterial pathogens, *e.g*. *Francisella tularensis* [3], [4], [14], [29], offers a promising clue. Macrophages–the main cellular target of *F. tularensis*—can develop the ability to kill the intracellular invaders after ‘activation’ by immune mediators, *e.g.* interferon-γ [13], [30], [31], [32]. Identification of the mechanisms and genes responsible for this beneficial transformation might provide a treasure trove of novel therapeutic targets, but this goal has not been met using traditional approaches.

The goal of this study was to identify novel therapeutic targets for infections caused by *F. tularensis.* The specific strategy was to use genome-wide shRNA interference to identify genes that mediate the beneficial, but poorly understood, effects of IFN-γ. The human macrophage cell line THP-1 provides a useful model for studies of *F. tularensis* intracellular pathogenesis, and reproduces the key response of interest, activation by IFN-γ of bacteriostatic/killing mechanisms for intracellular pathogens [7], [8], [9]. We used an optimized protocol to transduce THP-1 cells with the GeneNet™ Human 50K shRNA Library, and generated a library of THP-1 cells with a different shRNA expression construct in every cell. The main advantage of this strategy is the possibility of creating a very high complexity genome-wide shRNA library of all genes including some ESTs, with application of global loss-of-function genetic screen for unbiased discovery of genes involved in specific phenotypes.

Using this lentiviral shRNA-based loss-of-function screen for the THP-1 library, we were able to identify and sort IFN-γ-activated macrophages that failed to limit intracellular growth of green fluorescent *F. tularensis* [21]. Repeated screens and sorts allowed us to identify by sequencing the shRNAs which were enriched in multiple experiments (Table S1). These initial hits were further evaluated and validated by gene expression analysis, and functional testing using individual lentiviral constructs and siRNAs. Validation steps included use of different targeting sequences, since the possibility of off-target effects is well-known for RNAi. We were able to functionally replicate increased bacterial proliferation upon knockdown of the top 20 genes (Table 3). Our validation analysis also used human alveolar macrophage-like monocyte-derived macrophages and normal human lung macrophages to allow focus on genes functional in primary cells.

GFP-labeled *F. tularensis* Live Vaccine Strain was an ideal tool which permitted a strategy to do our screening and analysis under BL2 conditions. Although we used GFP-labeled *F. tularensis* LVS in our initial RNAi screen, most of a subset tested (5 of 6 genes) exhibited resistance to both LVS and a virulent *F. tularensis* strain (Fig. 5B and 5C, Fig. 7B). These data now support the value of undertaking future studies under more complex and demanding BL3 conditions. In addition, several top hits were tested with another intracellular pathogen, *Listeria monocytogenes*, and our results revealed that these IFN-γ induced beneficial genes (TNFRSF9, SERPINI1, SERPINA7, HLA-DRB1, ATG5, and ATG16L1) were also functional relevant to *Listeria* infection (Fig. 8), consistent with shared host response mechanisms to both intracellular pathogens, *F. tularensis* and *L. monocytogenes*. It will be interesting to test these gene functions in other intracellular pathogens including *Mycobacterium tuberculosis.*

We succeeded in identifying numerous novel genes involved in control of *F. tularensis* by activated macrophages. The results are the list of potential therapeutic targets tabulated in Table 3. Among the more promising of these are the top five targets shown in Table 4, which are considered to be ‘druggable’ [33], [34], [35], [36], [37] (http://www.openbiosystems.com/RNAi/shRNAmirLibraries/GIPZLentiviralshRNAmir/HumanDruggablesubset/) or potentially modulated by existing pharmacologic or therapeutic agents. In addition to providing the list of therapeutic targets, our research has established the utility of the functional screening approach for drug target identification in bioterrorism infectious agents. It is especially noteworthy that the top potential target gene product, TNFRSF9, is also known as CD137 [28], [38], [39], a receptor target that is presently being investigated in human clinical trials using a humanized monoclonal antibody that acts as an agonist. CD137 has been identified as a novel and potent monocyte activation factor [26], and it promotes adherence and prolongs survival of human peripheral monocytes by inducing a strong expression of macrophage colony-stimulating factor, an essential monocyte survival factor [28]. We found that CD137 knockdown with a blocking anti-CD137 antibody resulted in markedly increased bacterial load in human monocyte-derived macrophages (Fig. 9A–E), reproducing the phenotype that led to its identification in our screen. Ingenuity pathway analysis suggested that TNFRSF9/CD137 may interact with NF-κB, Akt, P38 MAPK and IL12 signaling pathways. Future studies with CD137 agonists are warranted to test their potential beneficial effect in *F. tularensis* infection, and more studies will be needed to elucidate the mechanism of CD137 clearance of *F. tularensis* in macrophages.

In summary, this genome-wide functional screen identified numerous novel genes that may help activated macrophages control intracellular replication of *Francisella*. Our validation efforts addressed a partial subset, and generally confirmed the initial findings of the RNAi screen. Although additional validation of the genes tabulated is needed, this data set provides ample leads for future investigation.

## Materials and Methods

### Reagents

Phorbol-12-myristate acetate (PMA), hexadimethrine bromide (Polybrene), penicillin/streptomycin, gentamicin, puromycin, ampicillin, human AB serum, MISSION® lentiviral transduction particles were purchased from Sigma-Aldrich, recombinant human interferon-gamma (IFN-γ) and human granulocyte/macrophage-colony stimulating factor (GM-CSF) from PeproTech, DNeasy Blood & Tissue Kit, DNaseI, RNeasy Mini Kit, siRNAs and HiPerFect transfection reagent from Qiagen, and PureLink™ Quick Gel Extraction Kit, PureLink™ Quick Plasmid Miniprep Kit, TOPO TA Cloning, TRIZOL reagent, and CellMask™ Blue and Hoechst 33342 dyes from Invitrogen (Life Technologies). RT^2^ First Strand kit, RT^2^ profiler™ PCR arrays, RT^2^ qPCR Primers, and RT^2^ SYBR® Green/ROX qPCR Master Mix were obtained from SABiosciences. Anti-CD137 mouse monoclonal (clone 4B4) was purchased from BioLegend, and mouse IgG_1_ isotype control antibodies from Sigma-Aldrich.

### Culture of macrophages

Human monocyte-like THP-1 cells were obtained from the American Type Culture Collection. THP-1 cells were cultured in RPMI complete medium (RPMI 1640 medium supplemented with 10% fetal bovine serum, 2 mM L-glutamine, 100 U/ml penicillin, 100 µg/ml streptomycin, 50 µM 2-β-mercaptoethanol, 1 mM sodium pyruvate) at 37°C in 5% CO_2_.

Human alveolar-like peripheral blood monocyte-derived macrophages (HMDMs) were prepared as previously described [25]. Primary human alveolar macrophages (AMs) were obtained by bronchoalveolar lavage from healthy human volunteers and cultured in RPMI complete media for 2 days before infection. Human cell samples were collected after obtaining written informed consent and were approved by institutional review boards at Harvard School of Public Health and Beth Israel Deaconess Medical Center, respectively.

### Transduction of THP-1 cells with shRNA library

We used the GeneNet™ Human 50 k shRNA Library from System Biosciences Inc (SBI) for this screen. The library contains ∼200,000 siRNA templates targeted to 47,400 human transcripts listed in the NCBI RefSeq database, packaged in a Feline Immunodeficiency Virus (FIV)-derived vector, pSIF1-H1-Puro. The packaged library was transduced into THP1 cells according to the manufacturer's recommendations. Four days after transduction, the number of shRNA constructs per macrophage was determined using SBI's Lentivector Rapid Titer PCR Kit, according to the manufacturer's protocol. GFP expression in THP1 cells transduced with a GFP reporter construct (pSIF1-H1 siLuc-copGFP, SBI) was used to construct a calibration standard for this assay. Transduced macrophages were selected with puromycin (1 µg/ml) for 9–10 days.

### Growth of *F. tularensis* and macrophage infection

*F. tularensis subsp. holoarctica* live vaccine strain (LVS) with plasmid pKK214GFP/ASV [21] coding for green fluorescent protein was generously provided by Dr. Larry S. Schlesinger (Ohio State University, Columbus, Ohio). Bacteria were grown to logarithmic phase in Modified Mueller-Hinton broth with tetracycline (10 µg/ml) and aliquots stored at −80°C. Before each infection experiment, the bacteria were thawed, streaked on Cystine Heart agar plates enriched with 1% hemoglobin and containing tetracycline for 1 day at 37°C. Bacteria were then suspended in PBS at an optical density of ∼0.405 at 600 nm, corresponding to 3×10^9^ bacteria/ml. Virulent *F. tularensis subsp. tularensis* strain SchuS4 was grown and used in the New England Regional Centre of Excellence in Biodefense and Emerging Infectious Diseases at Harvard Medical School. 24 h prior to infection, bacteria were streaked onto hemoglobin-enriched Cystine Heart agar plates and grown at 37°C. Bacteria were then suspended in PBS and concentration estimated by optical density. To increase uptake of *F. tularensis* by macrophages, both LVS and virulent strains were opsonized with 10% fresh human AB serum in PBS for 30 min at 37°C [21], [40], then diluted with RPMI medium containing 10% human AB serum to the desired MOI prior to infection. Macrophage infections were performed at the MOIs listed in the figure legends. Bacterial densities were confirmed by plating dilutions of inolcula and counting colonies.

THP-1 cells, HMDMs, and primary human AMs were subjected to the same general bacterial infection protocol, with some variations. THP-1 cells and THP-1 shRNA library cells were seeded in 6-well tissue culture plates at a density of 8×10^5^ cells per well in the presence of 100 nM PMA, and incubated for 24 hours. Medium containing PMA was then removed and cells were incubated for overnight in fresh medium with IFN-γ. HMDM cells and primary human AMs were seeded at 1×10^6^ cells/well in 6-well plates for 2 days prior to infection. At time of infection cells were washed with warm RPMI without antibiotics, and 2 ml of RPMI/10% human AB serum with opsonized *F. tularensis* was added to each well. Cells were infected at an MOI of ∼40∶1 in all screens. The plates were then centrifuged at 900× *g* for 20 minutes and incubated for 2 hours at 37°C with 5% CO_2_. Cells were then washed with fresh media, and incubated for 30 min at 37°C with media containing gentamicin (50 µg/ml) to kill any extracellular bacteria. Cells were washed again and cultured in RPMI/10% FBS medium for 24 hours at 37°C in 5% CO_2_.

### Culture of Listeria monocytogenes

*L. monocytogenes* strain 10403S expressing green fluorescent protein (s-gfp/pPL3) was generously provided by Dr. Darren E. Higgins (Harvard Medical School, Boston, MA). The *L. monocytogenes*-GFP were grown in brain heart infusion medium containing 7.5 µg/ml of chloramphenicol (GFP plasmid selection agent) for 15–18 h at 30°C without shaking. Macrophages were infected using a protocol similar to that described for *F. tularensis*.

#### CD137 antibody blocking F. tularensis phagocytosis by HMDMs

All reagents and buffers were at room temperature when added to cells, and all incubations were performed at 5% CO_2_ and 37°C, unless otherwise specified. HMDMs were suspended at 4×10^5^/ml in RPMI/10% FBS and dispensed into black-walled 96 well BD-Falcon Imaging plates (BD Biosciences) at 1.0×10^5^ cells/well, or 6-well cell culture plates at 1.0×10^6^ cells/well, and incubated for 24 h to become adherent. Cells were then incubated overnight with fresh RPMI/10% FBS containing either 5.0 µg/ml anti-CD137 antibody, 5.0 µg/ml IgG_1_ isotype control or no treatment. HMDMs were infected with suspensions of LVS-GFP, at 2.0×10^9^ CFU/ml in RPMI/0.3% BSA (total volume 100 µl/well for 96-well plates and 1.5 ml/well for 6-well plates) for 2 h, then incubated for 30 min with gentamicin in RPMI/10% FBS to kill extracellular bacteria, washed three times with PBS to remove gentamicin, incubated overnight with fresh RPMI/10% FBS containing treatments as detailed above, and processed as described below for flow cytometry, scanning cytometry, and CFU quantification.

### Flow cytometry

Human macrophages infected or uninfected with green fluorescent protein labeled bacteria were analyzed using a FacsCanto II flow cytometer (BD Biosciences). At the indicated time points post-infection, supernatants from 6-well plates were aspirated and HMDMs were harvested using 0.25% Trypsin-EDTA (to harvest THP-1, 0.01% Typsin-EDTA was used). The cells were spun down and fixed with 1.5% paraformaldehyde in PBS for 10 min, then washed once with PBS, and resuspended in PBS. 10000 cells were analyzed for each sample for green fluorescence.

### Scanning cytometry

Cells in black-walled 96-well plates were fixed by incubation with 4% formaldehyde for 10 minutes at room temperature, washed twice with PBS, incubated with HBSS/2.5 µg/ml HCS CellMask Blue cytoplasmic stain and 2 µg/ml Hoechst nuclear stain, washed twice with PBS, and finally covered with PBS. Confocal fluorescence microscopy was performed using a BD Pathway 855 High-Content Bioimager with a 20× NA075 objective. Collapsed confocal stack GFP channel images (GFP-expressing *F. tularensis*) were acquired using 470/40 band-pass (BP) excitation, 515 long-pass (LP) dichroic and 515/30 BP emission filters. Single confocal Hoechst nuclear/CellMask Blue cytoplasmic label images were acquired using 350/50 BP excitation, 400 LP dichroic, 435 LP emission filters. Sufficient image fields were collected from duplicate wells to provide a minimum of 500 cells for quantitative analysis of each sample condition. Image analysis and quantification was performed to quantify average number of bacteria/cell using our custom MATLAB® (The Mathworks, Natick, MA) software [41].

### Bacterial CFU assay

Macrophages were lysed in 1 ml (per well of a 6-well dish) of PBS containing 0.1% Saponin at room temperature for 10 min. Lysates were serially diluted in PBS, and 100 µl of each dilution was spread on Cystine Heart agar/1% Hemoglobin plates. The number of colonies was counted after 3 days' growth at 37°C, and used to calculate the number of bacteria in the original cell lysates.

### FACS sorting of target cells

Following transduction with the GeneNet™ human 50 K shRNA library, PMA-differentiation, IFN-γ-activation, and incubation with GFP-LVS for 24 h, THP-1 cells were harvested and sorted using the BD FACS Aria sorting flow cytometer in the Brigham & Women's Hospital Core Facility (Boston, MA). Appropriately 200 K to 300 K cells with the highest green fluorescence signal (top 1%) were collected from the pool of infected cells in each screen experiment (“sort”). Although it is possible that siRNAs may target GFP or bacterial gene expression without inhibiting bacteria, this is a concern for screens that rely on decreased expression; in contrast, our screen was designed to identify increased GFP content.

### Genomic DNA extraction and total RNA isolation

Total genomic DNA was isolated and purified from sorted target cells using the Qiagen DNeasy Blood and Tissue Kit. Approximately 1–4 µg total genomic DNA was obtained from each FACS sort. Total RNA was isolated using TRIzol Reagent. RNAs were further cleaned up by using RNeasy Mini kit with DNase I treatment to improve the quality of the final RNA samples.

### Identification of shRNA inserts by PCR Amplification, cloning and DNA sequencing

shRNA inserts from the target cells were amplified from genomic DNA (isolated from sorted cells) in two rounds of PCR using 2 sets of primers. The first round of PCR was performed using the primers provided with the shRNA library kit from SBI, referred to as the Fwd GNF Primer and the Rev GNF Primer. The second round of PCR was performed using primers referred to as Fwd 37: 5′-CGTGAAAT GTCTTTGGATTTGGG-3′, and SBI's Rev Nested Primer: 5′-AAAGAATGCTTATGGACGCTAGA-3′. The products from the first round of PCR were used as the templates for the second round. After the second round of PCR, the products were run on a 2.5% agarose gel and purified using the PureLink Quick Gel Extraction Kit. The purified PCR products were then cloned into competent *E. coli* DH5α-T1R using the TOPO TA Cloning Kit. White clones grown on LB/Ampicillin (100 µg/ml) and X-gal selective plates were picked for DNA sequencing at the Dana Farber/Harvard Cancer Center DNA Resource Core. DNA sequences were analyzed using Sequencer 4.8 software from Gene Codes Corporation. This software allowed us to identify the shRNA inserts within sequence by comparing the known plasmid sequences on either side of the target shRNA inserts within the pSIF1-H1 vector. The shRNA target sequences were then subjected to a BLAT (http://www.genome.ucsc.edu/) search to identify the corresponding targeted genes based on sequence similarity. More than 70% of total genes/transcripts/ESTs were identified using BLAT search. In cases in which, as a result of the high stringency of the BLAT program (requiring over 90% identity), a gene was not found, the sequence was subjected to BLAST search (www.ncbi.nlm.nih.gov/blast), which matches genes to sequences with the closest match.

### Validation using PCR arrays

Expression of mRNA for the top 168 genes from our primary screen was evaluated using custom PCR arrays from SABioscience Inc, according to the manufacturer's recommended protocol, and using their software. Expression levels were obtained and evaluated for three macrophage types (THP-1, HMDMs, and primary AMs) under 4 conditions (with or without IFN-γ activation, and with or without *F. tularensis* LVS infection). Duplicate samples (in duplicate plates) were tested for each cell type and condition, and expression levels were normalized against those of housekeeping genes using the recommended algorithm. Expression level data were compared across the three cell types to identify gene candidates with shared expression for further functional validation. Heat maps showing 168 gene expression levels in macrophages with IFN-γ activation versus without IFN-γ induction were generated using Eisen Lab Cluster Analysis and Visualization Software (http://www.eisenlab.org/eisen/).

### Validation using lentiviral shRNA and siRNA

Each MISSION™ lentiviral particle pool (Sigma) consisted of an average of 3–5 individual shRNA constructs targeting different regions of each gene sequence. MISSION Non-target shRNA control transduction particles were used as a negative control. For stable expression experiments, THP-1 cells were plated at 5×10^5^/well in a 12-well plate in 1 ml of DMEM complete medium. Polybrene was added to the cells at a final concentration of 6 µg/ml, and viral particles were added at an MOI of 1. The cell-viral particle mixture was incubated at 37°C overnight followed the procedure provided by Sigma. On day 3, the medium was replaced with fresh complete medium containing 1 µg/ml puromycin for selection of transduced cells. Puromycin-containing medium was replaced every 3–4 days until resistant colonies could be identified. For each target gene at least 5 puromycin-resistant colonies were picked and expanded to be assayed for knockdown.

In transient expression experiments, PMA-differentiated THP-1 macrophages and HMDMs were transfected with FlexiTube siRNAs using HiPerFect transfection reagent (Qiagen). Ctr_AllStars_1 siRNA from Qiagen was used as a negative control. THP-1 cells were seeded at 400 K cells/well in 6-well plates in RPMI complete medium containing 100 nM PMA, and HMDM cells were seeded at 200 K cells/well in RPMI complete medium. Cells were incubated at 37°C in 5% CO_2_ for 24 hrs. Shortly before transfection, the culture medium was removed and replaced with 500 µl fresh RPMI complete medium, and the cells were returned to normal growth conditions. To create transfection complexes, 100 nM siRNA in RPMI without additives (total 500 µl) was incubated with 12 µl of HiPerFect transfection reagent for 10 minutes at room temperature. The complexes were added drop-wise onto the cells. Cells were incubated with the transfection complexes for 6–8 hours at 37°C in 5% CO2. 0.8 ml of RPMI complete medium was then added to each well and cells were incubated for 48 hours prior to bacterial infection assay as per above.

### Statistical analysis

All data were analyzed with Prism software 4.0 (GraphPad), using paired two-tailed Student's *t* tests to determine significance between groups unless otherwise specified. Differences between groups were considered statistically significant when the *P* value was <0.05. Unless otherwise indicated, at least three independent experiments were included in each analysis and the results were represented as means ± s.d.

## Supporting Information

Figure S1 **Optimizing transduction with lentiviral shRNA library.** Fluorescent microscopy imaging copGFP-positive THP-1 cells after transduction with pSIF1- H1-siLuc-copGFP (FIV-based) (A) or with pSIH1-copGFP (HIV-based) (B) packaged positive transduction control at viral titer 0, 3, 5, 10, 15 and 20. (C) Comparison of THP-1 cell transduction efficiency with FIV-based and HIV-based positive transduction control. Flow cytometry assay was performed to measure the % of GFP positive cells, which indicates the transduction efficiencies of THP-1. (D) Determining the % of THP-1 cells infected with GeneNet_TM_ Human 50 k siRNA library. MOI (multiplicity of infection) is the average copy number of lentiviral expression constructs per infected cell. The MOI in transduced cells was determined by comparison of WPRE gene expression in transduced THP-1 library cells with the Calibration Standard (MOI) using Lentivector Rapid Titer PCR kit. **The table was provided by SBI based on the percentage of GFP-positive cells in their results to determine the MOI.
(TIF)

Figure S2 **Functional categories of top 212 hits using Ingenuity pathway analysis.** The numbers on the pie charts represent the number of target genes within each functional category. (***a***) 110 Hits in Molecular and Cellular Functions. (***b***) 135 Hits in Diseases and Disorders. (***c***) 52 Hits in Physiological System Development and Function.
(TIF)

Table S1 Results of flow cytometric screening are tabulated according to the number of sorts and the number of clones in which a target gene was identified by sequencing. The table lists results for the 247 gene targets identified in two or more screens of the total of five conducted.
(PDF)
