# Supplementary figures and images for: Genome-Wide RNAi Screen in IFN-γ-Treated Human Macrophages Identifies Genes Mediating Resistance to the Intracellular Pathogen Francisella tularensis
Source: PLoS One. 2012 Feb 16;7(2):e31752. doi: 10.1371/journal.pone.0031752 (PMC3281001; doi:10.1371/journal.pone.0031752)

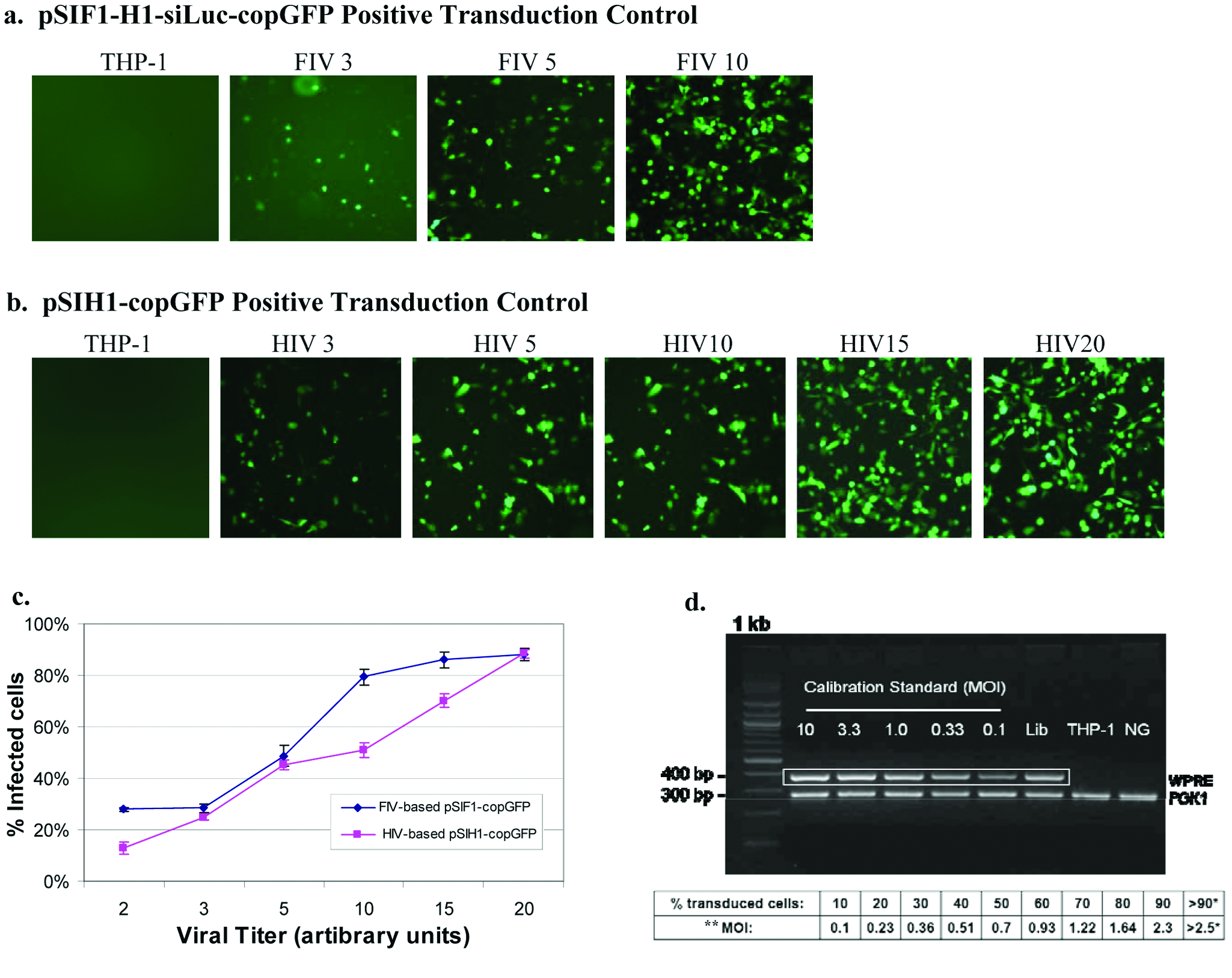

Supplement: Figure S1 — Optimizing transduction with lentiviral shRNA library. Fluorescent microscopy imaging copGFP-positive THP-1 cells after transduction with pSIF1- H1-siLuc-copGFP (FIV-based) (A) or with pSIH1-copGFP (HIV-based) (B) packaged positive transduction control at viral titer 0, 3, 5, 10, 15 and 20. (C) Comparison of THP-1 cell transduction efficiency with FIV-based and HIV-based positive transduction control. Flow cytometry assay was performed to measure the % of GFP positive cells, which indicates the transduction efficiencies of THP-1. (D) Determining the % of THP-1 cells infected with GeneNetTM Human 50 k siRNA library. MOI (multiplicity of infection) is the average copy number of lentiviral expression constructs per infected cell. The MOI in transduced cells was determined by comparison of WPRE gene expression in transduced THP-1 library cells with the Calibration Standard (MOI) using Lentivector Rapid Titer PCR kit. **The table was provided by SBI based on the percentage of GFP-positive cells in their results to determine the MOI. (TIF) [file pone.0031752.s001.tif]

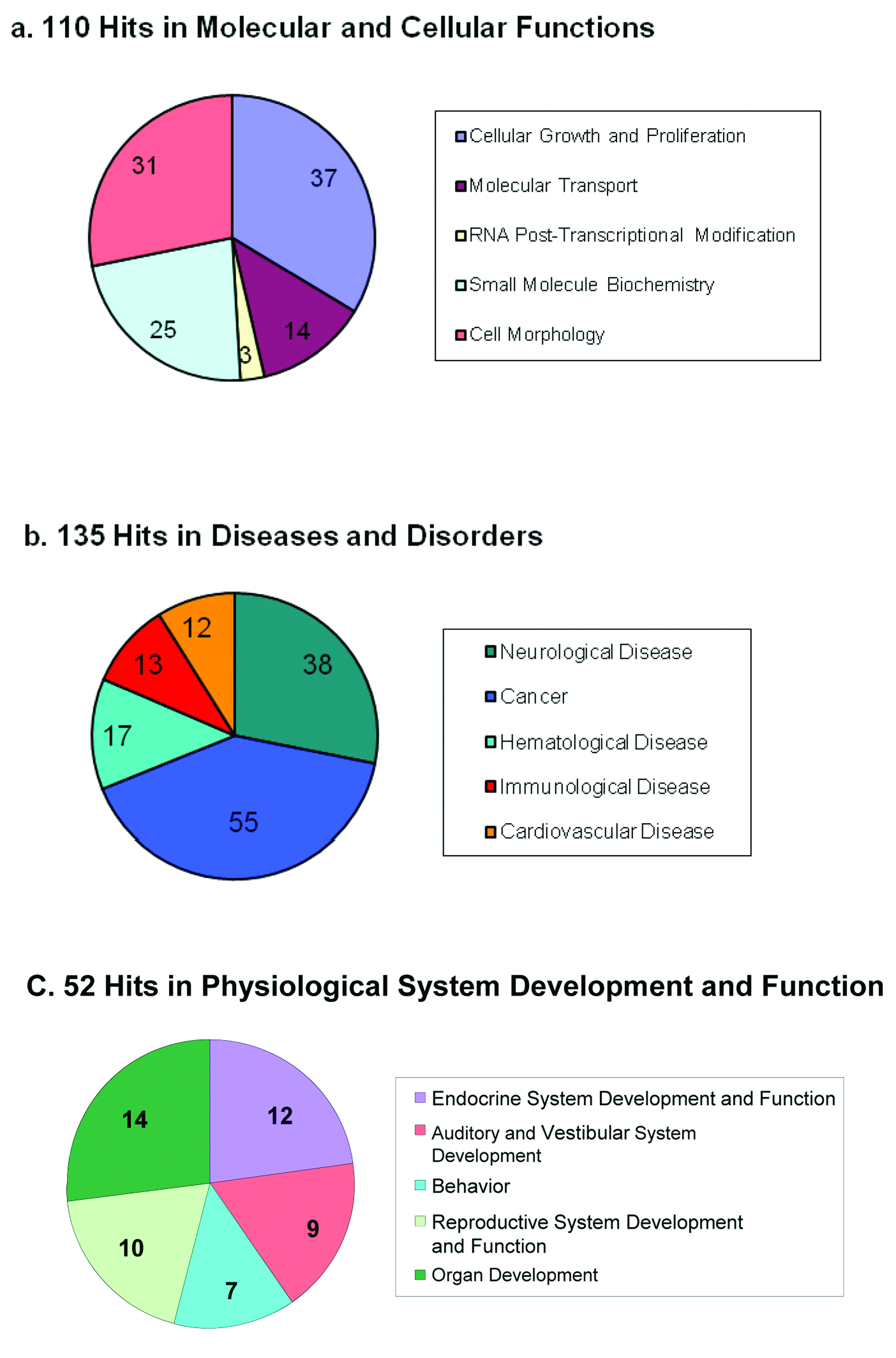

Supplement: Figure S2 — Functional categories of top 212 hits using Ingenuity pathway analysis. The numbers on the pie charts represent the number of target genes within each functional category. (a) 110 Hits in Molecular and Cellular Functions. (b) 135 Hits in Diseases and Disorders. (c) 52 Hits in Physiological System Development and Function. (TIF) [file pone.0031752.s002.tif]
