# Supplementary material for: Genome-Wide RNAi Screen in IFN-γ-Treated Human Macrophages Identifies Genes Mediating Resistance to the Intracellular Pathogen Francisella tularensis
Source: PLoS One. 2012 Feb 16;7(2):e31752. doi: 10.1371/journal.pone.0031752 (PMC3281001; doi:10.1371/journal.pone.0031752)
Supplement: Table S1 — Results of flow cytometric screening are tabulated according to the number of sorts and the number of clones in which a target gene was identified by sequencing. The table lists results for the 247 gene targets identified in two or more screens of the total of five conducted. (PDF) [file pone.0031752.s003.pdf]

Table S1. 247 Genes Identified in Multiple Hits (Top 212 genes identified in multiple sorts)

Gene and Function	EntrezGene ID	#Clones	#Sorts
**SERPINI1/SERPINB7/SERPINB6/SERPINA7/SERPINB2/SERPINB4:** serine (or cysteine) proteinase inhibitor.	NM_001122752.1	8	4
**TRIM10:** tripartite motif-containing 10 isoform 1 and 2. Seems to play an important role in erythropoiesis.	NM_006778.3	4	4
**ADAM9:** ADAM metallopeptidase domain 9 (meltrin gamma)	NM_001005845.1	4	3
**APC:** adenomatous polyposis coli. Antagonist of the Wnt signaling pathway.	NM_000038	5	3
**CASP8:** caspase 8 isoform B precursor. Caspases play a central role in the execution-phase of cell apoptosis.	NM_001228	4	3
**CCL8**: small inducible cytokine A8 precursor. This cytokine displays chemotactic activity for monocytes, lymphocytes, basophils and eosinophils.	NM_005623.2	3	3
**DICER1:** dicer 1, ribonuclease type III; This encoded protein possessing an RNA helicase motif containing a DEXH box in its amino terminus.	NM_177438	4	3
**EIF2AK2:** eukaryotic translation initiation factor 2-alpha kinase 2.	NM_002759.1	3	3
**EML1:** echinoderm microtubule associated protein like 1.	NM_001008707.1	4	3
**ENPP4:** ectonucleotide phyrophosphatase/phosphodiesterase 4 (putative function).	NM_014936.4	5	3
**FAF1**: Fas (TNFRSF6) associated factor 1 (UCSC); Mediates apoptosis in a number of organ systems.	NM_007051.2	7	3
**GCSH:** glycine cleavage system protein H (aminomethyl carrier)	NM_004483.3	8	3
**GDNF:** glial cell derived neurotrophic factor isoform 2.	NM_000514.2	3	3
**HNRPH2:** heterogeneous nuclear ribonucleoprotein H2. Influence pre-mRNA processing and other aspects of mRNA metabolism and transport.	NC_007878.1	5	3
**ITPR1/ITPR2/ITPR3:** inositol 1,4,5-triphosphate receptor, type 3	NM_001099952.1	3	3
**KL:** klotho isoform a. type-I membrane protein that is related to beta-glucosidases. Klotho-deficient mice had decreased nitric oxide production	NM_004795.2	5	3
**KLF11:** Kruppel-like factor 11. Transcription factor	NM_003597	3	3
**MED6:** Homo sapiens RNA polymerase II transcriptional regulation mediator (Med6, S. cerevisiae, homolog of), mRNA.	NM_005466	3	3
**MLLT10:** myeloid/lympohoid or mixed-lineage leukemia, 10.	NM_001009569.1	3	3
**NPY5R**: neuropeptide Y receptor Y5. Receptor for CCL2, CCL5, CCL8.	NM_006174	4	3
**PDCD4:** programmed cell death 4 isoform 1. Gene expression is modulated by cytokines in natural killer and T cells.	NM_014456.3	3	3
**PSIP1 / SNAPC3:** PC4 and SFRS1 interacting protein 1 isoform 2. Transcriptional coactivator.	NM_033222	4	3
**RECQL:** RecQ protein-like isoform 1. Unknown function, belongs to a DNA helicase family.	NM_002907	4	3
**SCGB2A2:** secretoglobin, family 2A, member 2	NM_002411	4	3
**SEMA3G:** semaphorin sem2	NM_020163	3	3
**SH2B3:** lymphocyte adaptor protein.	NM_005475	3	3
**SLC11A2:** solute carrier family 11 (proton-coupled). The product of this gene transports divalent metals and is involved in iron absorption.	NM_000617	2	3
**SLC25A46/SLC25A30/SLC25A16/SLC25A13:** solute carrier family 25, member 46. Mitochondrial carrier protein family.	NM_138773.1	4	3
**SLC4A7/SLC4A4/SLC4A8:** solute carrier family 4, sodium bicarbonate	NM_003615	5	3
**TCF7:** transcription factor 7 (T-cell specific)	NM_003202.2	3	3
**TEP1:** telomerase-associated protein 1, essential for the replication of chromosome termini.	NM_007110.3	7	3
**TSHR:** thyroid stimulating hormone receptor.	NM_000369.2	3	3
**TTC9:** tetratricopeptide repeat domain 9	NM_015351.1	3	3
**ZFR:** zinc finger RNA binding protein.	NM_016107	3	3
**ZNF193:** zinc finger protein 193. May be involved in transcriptional regulation.	NM_006299	4	3
**ZNF228 (ZNF112):** zinc finger protein 228 or 112	NM_00108335.1	4	3
**ZNF638:** zinc finger protein 638. Binds ctyidine-rich sequences in dsDNA. It is associated with packaging, transferring, or processing transcripts.	NM_014497	3	3
**ABCC4:** ATP-binding cassette, sub-family C, member 4.Transports various molecules across extra- and intra-cellular membranes.	NM_001105515.1	2	2
**ABCC9:** ATP-binding cassette, sub-family C, member 9. ABC proteins transport various molecules across extra- and intra-cellular membranes	NM_005691.2	3	2
**ABHD3:** alpha/beta hydrolase domain containing protein. Function unknown.	NM_138340	2	2

Gene and Function	EntrezGene ID	#Clones	#Sorts
**ACSL4:** acyl-CoA synthetase long-chain family member 4. Plays a key role in lipid biosynthesis and fatty acid degradation.	NM_004458.2	4	2
**ACTR2:** actin-related protein 2 isoform a. Specific function unknown.	NM_001005386	2	2
**ADAM30:** ADAM metallopeptidase domain 30 preproprotein. Membrane-anchored protein related to snake venom disintegrins. Testis-specific.	NM_021794.2	3	2
**ADAMTS1:** ADAM metallopeptidase with thrombospondin type 1. Expression may be associated with various inflammatory processes.	NM_006988.3	2	2
**ADD3:** adducin 3 (gamma) isoform a. Associated with the regulation of blood pressure.	NM_019903	2	2
**AGPS:** Homo sapiens alkylglycerone phosphate synthase, mRNA.	NM_003659	2	2
**AIFM1:** apoptosis-inducing factor, mitochondrion-associated, 1.	NM_145812	2	2
**ALDH2:** aldehyde dehydrogenase 2 family (mitochondrial), nuclear gene encoding mitochondrial protein.	NM_000690	3	2
**ANAPC2**: anaphase-promoting complex subunit 2. Promotes metaphase-anaphase transition.	NM_013366	3	2
**ANK1:** ankyrin 1 isoform 5. Believed to link the integral membrane proteins to the underlying spectrin-actin cytoskeleton.	NM_020475	3	2
**AP2S1:** adaptor-related protein complex 2, sigma 1. Associated with plasma membrane	NM_004069	2	2
**AQP4:** aquaporin 4 isoform a. membrane protein that functions as water-selective channels in the plasma membrane of many cells.	NM_001650	3	2
**ARL4:** ADP-ribosylation factor-like 4	NM_005738	2	2
**ATP2B4:** plasma membrane calcium ATPase 4 isoform 4b. Removes bivalent calcium ions from eukaryotic cells and create calcium homeostasis.	NM_001001396	2	2
**ATXN3:** ataxin 3 isoform 2.	NM_004993	2	2
**AZIN1:** ornithine decarboxylase antizyme inhibitor. Regulates cellular polyamine homeostasis.	NM_015878	2	2
**BAIAP2**: BAI1-associated protein 2 isoform 1. brain-specific angiogenesis inhibitor (BAI1)-bidning protein.	NM_006340	3	2
**BCHE:** butyrylcholinesterase.	NM_000055	2	2
**BDNF:** brain-derived neurotrophic factor isoform c. Member of nerve growth factor family. Necessary for survival of striatal neurons in the brain.	NM_170735	2	2
**BIRC3:** baculoviral IAP repeat-containing protein 3. Inhibits apoptosis by binding to tumor necrosis factor receptor-associaed factors.	NM_001165.3	2	2
**BNC1:** basonuclin 1. zinc finger protein present in the basal cell layer of epidermis and in hair follicles.	NM_001717	2	2
**BRCA1:** breast cancer 1, early onset isoform 1. Maintains genomic stability and acts as a tumor suppressor.	NM_007294	2	2
**C1orf9:** chromosome 1 open reading frame 9 protein	NM_016227	2	2
**CACNA1D**: calcium channel voltage-dependent L type	NM_000720	2	2
**CALM1**: calmodulin 1 (phosphorylase kinase, delta). Calmodulin mediates the control of a large number of enzymes and other proteins by Ca(2+).	NM_006888	7	2
**CD58:** CD58 molecule. Lymphocyte function-associated antigen 3. Cell adhesion molecule, mediates adhesion between killer and target cells, etc.	NM_001779	5	2
**CDC42EP4:** CDC42 effector protein (Rho GTPase binding) 4.	NM_012121	2	2
**CDH7:** cadherin 7, type 2 preproprotein. Calcium dependent cell-cell adhesion glycoprotein.	NM_033646	5	2
**CEACAM8:** carcinoembryonic antigen-related cell adhesion molecule 8.	NM_001816	2	2
**CFTR:** cystic fibrosis transmembrane conductance. Chloride channel.	NM_000492	3	2
**CHN2:** beta chimerin isoform 1. GTPase-activating protein activity. Plays a role in proliferation and migration of smooth muscle cells.	NM_004067	3	2
**Chromosome 2 genomic contig.** Near here is SRY-box 11 (SRY-box is gene SOX4)		4	2
**CLNS1A:** chloride channel, nucleotide-sensitive, 1A	NM_001293	3	2
**COL8A1:** alpha 1 type VIII collagen precursor. Major component of the basement membrane of the corneal endothelium.	NM_001850	2	2
**CRY1:** cryptochrome 1 (photolyase-like).	NM_004075	3	2
**CRYGD:** crystallin, gamma D.	NM_006891	2	2
**CSH1:** chorionic somatomammotropin hormone 1 (placental actogen)	NM_001317	4	2
**CSRP2:** cyteine and glycine-rich protein 2. May be involved in regulatory processes important for development and cellular differentiation.	NM_001321	2	2
**CUL4B:** cullin 4B.	NM_003588	3	2
**DFNA5:** Homo sapiens deafness, autosomal dominant 5, mRNA.	NM_004403	3	2
**DKC1:** dyskerin. Involved in various aspects of rRNA processing and modification.	NM_001363	3	2

Gene and Function	EntrezGene ID	#Clones	#Sorts
**DMXL2**: Dmx-like 2	NM_015263.2	2	2
**EBAG9:** estrogen receptor binding site associated. Estrogen-responsive gene.	NM_198120	3	2
**EEF1D:** eukaryotic translation elongation factor 1 delta. Responsible for enzymatic delivery of aminoacyl tRNAs to the ribosome.	NM_032378	3	2
**EGFR:** epidermal growth factor receptor isoform d. This protein is a receptor for members of the epidermal growth factor family.	NM_005228	3	2
**EHMT2:** HLA-B associated transcript 8 isoform a. Thought to be involved in intracellular protein-protein interaction.	NM_006709	2	2
**ELAVL2:** ELAV embryonic lethal, abnormal vision.	NM_004432	2	2
**EP300:** E1A binding protein p300. Functions as histone acetyltransferase and regulates transcription via chromatin remodeling.	NM_001429	2	2
**F2RL1**: H. sapiens coagulation factor II (thrombin) receptor-like 1	NM_005242.3	2	2
**F7:** coagulation factor VII isoform b precursor. Vitamin K-dependent factor essential for hemostasis.	NM_001034806	2	2
**FABP4:** fatty acid binding protein 4, adipocyte	NM_001442	3	2
**FBXO22:** F-box only protein 22 isoform a.	NM_147188	2	2
**FGF20:** fibroblast growth factor 20. Posesses broad mitogenic and cell survival activities.	NM_019851	2	2
**FGF7:** fibroblast growth factor 7 precursor. Growth factor active on keratinocytes.	NM_002009	3	2
**FLG:** filaggrin. Profilaggrin is a major protein component of the keratohyalin granules of mammalian epidermis.	NM_002016	2	2
**FLRT3**: fibronectin leucine rich transmembrane protein 3. Function in cell adhesion and/or receptor signaling.	NM_013281	3	2
**FXN:** frataxin isoform 1 preproprotein. Regulates mitochondrial iron transport and respiration.	NM_000144	6	2
**GAD2:** glutamate decarboxylase 2. Catalyzes the production of gamma-aminobutyric acid from L-glutamic acid.	NM_000818	3	2
**GAS7**: growth-arrest-specific 7 isoform b. Expressed primarily in brain cells. Plays a role in neural development.	NM_003644	4	2
**GFAP:** glial fibrillary acidic protein. One of the major intermediate filament proteins of mature astrocytes.	NM_002055	2	2
**GGH:** Homo sapiens gamma-glutamyl hydrolase (conjugase, folylpolygammaglutamyl hydrolase), mRNA.	NM_003878	2	2
**GHITM:** growth hormone inducible transmembrane protein.	NM_014394	2	2
**GPX3:** Homo sapiens glutathione peroxidase 3 (plasma), mRNA.	NM_002084	2	2
**GRHPR:** glyoxylate reductase/hydroxypyruvate reductase. Role in metabolism.	NM_012203	2	2
**GRM1:** glutamate receptor, metabotropic 1. G protein-coupled receptors, inbolbed in most aspects of normal brain function.	NM_000838	3	2
**HIVEP1:** human immunodeficiency virus type I enhancer. Bind specific DNA sequences in the promoters.	NM_002114	3	2
**HLA-DRB1/DRB4:** MHC class II HLA-DR beta 1 or 4 chain..	NM_002124	2	2
**HNRPA3:** heterogeneous nuclear ribonucleoprotein A3	NM_194247	2	2
**Homo sapiens hypothetical protein FLJ22670**	NM_025144	2	2
**Homo sapiens OKSW-cl.89 mRNA, complete cds.**		2	2
**HSD3B2:** hydroxy-delta-5-steroid dehydrogenase, 3 beta. Crucial in biosynthesis of all classes of hormonal steroids.	NM_000198	2	2
**HTATIP2:** oxidoreductase (HIV-1 TAT-interactive protein 2)	NM_001098521	2	2
**Hypothetical protein LOC100132444**		4	2
**IFNB1**: inerferon, beta 1, fibroblast.	NM_002176	2	2
**ITGA6/ITGA5:** integrin alpha chain 5 or 6.	NM_000210	2	2
**KATNB1:** katanin p80 (WD repeat containing) subunit B 1.	NM_005886	3	2
**KCND2**: potassium voltage-gated channel, Shal-related. Prominent in the repolarization phase of the action potential.	NM_012281	4	2
**LCP2:** lymphocyte cytosolic protein 2.	NM_005565	3	2
**LEPR:** leptin receptor isoform 1. Regulates adipose-tissue mass through hypothalamic effects on satiety and energy expenditure.	NM_002303	5	2
**LGI1:** leucine-rich, glioma inactivated 1.	NM_005097	2	2
**LHCGR:** Homo sapiens luteinizing hormonechoriogonadotropin receptor, mRNA.	NM_000233	6	2

Gene and Function	EntrezGene ID	#Clones	#Sorts
**LRBA:** LPS-responsive vesicle trafficking.	NM_006726	3	2
**LRRFIP2:** leucine rich repeat (in FLII) interacting. May function as activator of the canonical Wnt signaling pathway, in associate with DVL3.	NM_006309	3	2
**M6PRBP1:** mannose 6 phosphate receptor binding protein 1.	NM_005817	2	2
**MAP4K4:** mitogen-activated protein kinase kinase kinase. Shown to specifically activate MAPK8/JNK.	NM_004834	4	2
**MBNL1/MBNL3:** muscleblind-like 1 or 3	NM_021038, NM_018388	2	2
**MCL1:** myeloid cell leukemia sequence 1 isoform 2. This gene encodes an anti-apoptotic protein, which is a member of the Bcl-2 family.	NM_021960	2	2
**MED12:** mediator of RNA polymerase II transcription.	NM_005120	4	2
**METTL9:** methyltransferase like 9 isoform 2	NM_016025	2	2
**MPZL1:** Homo sapiens myelin protein zero-like protein MPZL1a mRNA, complete cds.	NM_024569	2	2
**MTDH:** LYRIC/3D3 (Lysine-rich CEACAM1 co-isolated protein). Metastasis adhesion protein. Potential mediator of lung metastasis.	NM_178812	2	2
**MTF2:** metal response element binding transcription; Putative DNA binding protein	NM_007358	4	2
**MYBPC1**: myosin binding protein C, slow type, isoform 4	NM_002465	2	2
**NAG:** neuroblastoma-amplified protein	NM_015909	2	2
**NAP1L2:** nucleosome assembly protein 1-like 2. Interacted with chromatin to regulate neuronal cell proliferation.	NM_021963.2	2	2
**NBEA:** neurobeachin. Binds to type II regulatory subunits of protein kinase A and anchors/targets them to the membrane	NM_015678	3	2
**NCBP1:** nuclear cap binding protein subunit 1. Promotes high-affinity mRNA-cap binding and associates with the CTD of RNA polymerase II.	NM_002486	3	2
**NDUFA9:** NADH dehydrogenase (ubiquinone) 1 alpha subcomplex, 9	NM_005002	3	2
**NFE2L1:** nuclear factor (erythroid-derived 2)-like 1. Involved in globin gene expression in erythrocytes.	NM_003204	5	2
**NMT2:** N-myristoylransferase 2. catalyzes the reaction of N-terminal myristoylation of many signaling proteins.	NM_004808	2	2
**NRP1:** neuropilin 1. Plays versatile roles in angiogenesis, axon guidance, cell survival, migration, and invasion.	NM_003873	2	2
**NUFIP1:** nuclear fragile X mental retardation protein. Binds RNA	NM_012345	2	2
**PARVA:** parvin, alpha. Actin-binding proteins associated with focal contacts.	NM_018222	3	2
**PCDHA1:** protocadherin alpha 1 isoform 1 precursor. Critical role in establishment and function of specific cell-cell connections in the brain.	NM_018900	3	2
**PCOLCE2:** procollagen C-endopeptidase enhancer 2	NM_013363	3	2
**PDCL:** phosducin-like. Putative modulator of heterotrimeric G proteins.	NM_005388	3	2
**PDGFRA:** platelet-derived growth factor receptor, alpha polypeptide.	NM_006206	2	2
**PDHX:** pyruvate dehydrogenase complex, component X.	NM_003477	4	2
**PDK4:** pyruvate dehydrogenase kianse 4. Contributes to the regulation of glucose metabolism.	NM_002612	3	2
**PEX1:** peroxin 1. Encodes a member of the AAA ATPase family. This protein is cytoplasmic but often anchored to a peroxisomal membrane.	NM_000466	3	2
**PEX11A:** peroxisomal biogenesis factor 11A	NM_003847.1	4	2
**PFAAP5:** phosphonoformate immuno-associated protein 5.	NM_014887	2	2
**PGGT1B:** protein geranylgeranyltransferase type I, beta subunit.	NM_005023	2	2
**PICALM:** phosphatidylinositol-binding clathrin assembly.	NM_007166	2	2
**PLCB2:** phospholipase C, beta 2.	NM_004573	2	2
**PLS1:** plastin 1. family of actin-binding proteins. AKA Fimbrin,	NM_002670	2	2
**PLXNC1:** plexin C1. Receptor for vaccinia virus sempaphorin A39R and herpesvirus Sema protein.	NM_005761	2	2
**POLQ:** polymerase (DNA directed), theta	NM_199420	2	2
**POU4F2:** Brn3b POU domain transcription factor. Observed to play impt. Roles in control of cell identity in several systems.	NM_004575	2	2
**PRF1:** perforin 1 precursor. Key effector molecule for T-cell and natural killer-cell-mediated cytolysis.	NM_005041	2	2
**PTBP2:** polypyrimidine tract binding proten 2.	NM_021190	3	2

Gene and Function	EntrezGene ID	# Clones	#Sorts
**PTPRA:** protein tyrosine phosphatase (PTP), receptor type, A. This PTP dephosphorylates and activates Src family tyrosine kinases.	NM_002836.3	2	2
**PUM2:** pumilio (Drosophila) homolog 2	NM_015317	2	2
**RAB11FIP3/** RAB11FIP2: rab11-family interacting protein 2 or 3. Acts as a regulator of endocytic traffic by participating in membrane delivery**.**	NM_014700	2	2
**RAPGEF2:** Rap guanine nucleotide exchange factor 2.	NM_014247	2	2
**RARRES1:** retinoic acid receptor responder (tazarotene). Upregulated by tazarotene and retinoic acid receptors.	NM_002888.2	2	2
**RCC1:** Homo sapiens chromosome condensation 1-like, mRNA.	NM_001268.2	3	2
**SYT1:** synaptotagmin I. May serve as Ca(2+) sensors in the process of vesicular trafficking and exocytosis.	NM_005639	2	2
**RFC5:** Homo sapiens, replication factor C (activator 1) 5 (36.5kD), clone MGC:1155, mRNA, complete cds.	NM_007370	2	2
**RGS10:** regulator of G-protein signaling 10. Act as GTPase activating proteins for G alpha subunits of heterotrimeric G proteins**.**	NM_002925	2	2
**RPL31:** RPL31 protein. This protein bleongs to the L31E family of ribosomal proteins, located in the cytoplasm.	NM_001098577	2	2
**RYBP:** YEAF1 mRNA for YY1 and E4TF1 associated factor 1	NM_012234	3	2
**SCAMP1:** secretory carrier membrane protein 1. Carriers to the cell surface in post-golgi recycling pathways.	NM_004866	4	2
**SCN3B:** sodium channel, voltage-gated, type III, beta	NM_018400	2	2
**SCN4A:** voltage-gated sodium channel type 4 alpha. Responsible for the generation and propagation of action potentials in neurons and muscle.	NM_000334	3	2
**SEC24D:** Sec24-related protein D. Involved in vesicle trafficking.	NM_014822.2	2	2
**SEC63:** SEC63 homolog (S. cerevisiae). Central component of the protein translocation apparatus of the ER membrane.	NM_007214	4	2
**SFRP1:** secreted frizzled-related protein 1. Soluble modulator of Wnt signaling.	NM_003012	2	2
**SFRS5:** splicing factor, arginine/serine rich 5. Plays a role in constitutive splicing and can modulate the selection of alternative splice sites.	NM_001039465	2	2
**SH3BP5:** SH3-domain binding protein 5 (BTK-associated)	NM_004844	2	2
**SHC4:** rai-like protein / **EID1:** CREBBP/EP300 inhibitor 1.	NM_203349	2	2
**SIRT4**: sirtuin 4. unknown function	NM_012240	2	2
**SLC19A3:** solute carrier family 19, member 3. Part of reduced folate family of micronutrient transporter genes.	NM_025243	2	2
**SLC26A4:** pendrin. Sodium-independent transporter of chloride and iodide.	NM_000441	2	2
**SSX3:** synovial sarcoma, X breakpoint 3 isoform a. May function as transcriptional repressors.	NM_021014	3	2
**STRN3:** nuclear autoantigen. Binds calmodulin in a calcium dependent manner. May function as scaffolding or signaling protein.	NM_014574	4	2
**STX3:** syntaxin 3. Potentially involved in docking of synaptic vesicles at presynaptic active zones.	NM_004177	2	2
**STXBP5L:** syntaxin-binding protein 5-like (Tomosyn-2). May play a role in vesicle trafficking and exocytosis.	NM_014980	2	2
**SULT1C2:** sulfotransferase family, cytosolic, 1C, member 2. Catalyzes the sulfate conjugation of many drugs, hormones and neurotransmitters**.**	NM_001056.3	8	2
**SYNC1:** Syncoilin, intermediate filament 1	NM_030786	4	2
**TAOK1:** TAO kinase 1. Phosphorylates MKK3 (by similarity). Activates the p38 MAP kinase pathway.	NM_020791	9	2
**TBL1X:** transducin beta-like 1X. Plays an essential role in transcription activation mediated by nuclear receptors.	NM_005647	2	2
**TBX4**: T-box. Involved in the transcriptional regulation of genes required for mesoderm differentiation.	NM_018488	2	2
**TFPI2:** tissue factor pathway inhibitor 2.	NM_006528	2	2
**TFRC:** transferrin receptor	NM_003234	2	2
**TGOLN2:** trans-golgi network protein 2.	NM_006464	4	2
**THRB:** thyroid hormone receptor, beta (erythroblastic leukemia viral oncogene homolog 2, avian)	NM_000461	2	2
**TIA1:** TIA1 protein isoform 1. Member of RNA-binding protein family and possesses nucleolytic activity.	NM_022037	2	2
**TLL2:** tolloid-like 2. Member of metzincin family.	NM_012465	4	2
**TM2D2:** TM2 domain containing 2 isoform b. May have a regulatory roles in cell death or proliferation signal cascades.	NM_031940	2	2
**TNFRSF9:** tumor necrosis factor receptor superfamily 9. The receptor contributes to the clonal expansion, survival, and development of T cells.	NM_001561	2	2

Gene and Function	EntrezGene ID	#Clones	#Sorts
**RIB3: t**ribbles 3, protein kinase domains containing protein similar to phosphoprotein C8FW (LOC57761), mRNA.	NM_021158	3	2
**TRIM49:** ring finger protein 18.	NM_020358	4	2
**TRPA1:** transient receptor potential cation channel, ankyrin-like protein 1. May function in signal transduction and growth control.	NM_007332	3	2
**TUBB1/TUBB2C/TUBB6:** Tubulin, beta, class VI (1)/beta 2 (2C)/beta 6 (6)	NM_030773	8	2
**UBLCP1:** ubiquitin-like domain containing CTD phosphatase.	NM_145049	2	2
**UBXD2:** integral membrane protein of the ER that binds valosin-containing protein and promotes ER-associated protein degradation.	NM_014607	2	2
**UGT2B17**: Human UDP glucuronosyltransferase mRNA, partial cds.	NM_001077	4	2
**WIF1:** WNT inhibitory factor 1 precursor. Binds WNT proteins and inhibits their activities (control of embryonic development).	NM_007191	2	2
**ZNF263:** zinc finger protein 263	NM_005741	2	2
**ZNF639:** zinc finger protein 639	NM_016331	2	2
**ABHD2:** alpha/beta hydrolase domain containing protein. Function unknown.	NM_152924	1	1
**ADD2**: H. sapiens adducin 2 (beta) , transcript variant beta-4, mRNA.	NM_001617	2	1
**ATP6V1D:** H+ transporting two-sector ATPase.mediates acidification of intracellular organelles	NM_015994	2	1
**BAG4:** BCL2-associated athanogene 4. Inhibits the chaperone activity of HSP70/HSC70 by promoting substrate release.	NM_004874	2	1
**CACNA1S:** calcium channel, voltage-dependent, L type. Slowly activating, skeletal muscle.	NM_000069	2	1
**CSNK1G2**: casein kinase 1, gamma 2	NM_001319	2	1
**CYP1B1:** cytochrome P450, family 1. Cytochromes P450 are a group of heme-thiolate monooxygenases.	NM_000104	2	1
**CYP3A43**: cytochrome P450, family 3, subfamily A, polypeptide 43.	NM_057095	2	1
**DDX18:** DEAD (Asp-Glu-Ala-Asp) box polypeptide 18.	NM_006773	2	1
**DMXL1:** Dmx-like 1. Unknown function.	NM_005509	3	1
**DUSP1:** dual specificity phosphatase 1. Expression is induced in human skin fibroblasts by oxidative/heat stress and growth factors.	NM_004417	2	1
**EIF4G2:** eukaryotic translation initiation factor 4 gamma, 2	NM_002759	2	1
**EMP1:** epithelial membrane protein 1.	NM_001423	2	1
**GABRA2:** gamma-aminobutyric acid A receptor, alpha 2.	NM_000807.2	2	1
**GALNT2:** polypeptide N-acetylgalactosaminyltransferase 2.	NM_004481	1	1
**GNG13:** guanine nucleotide binding protein (G protein). Involved as a modulator or transucer in various transmembrane signaling systems.	NM_016541	3	1
**GSTM2:** Homo sapiens glutathione S-transferase M2 (muscle), mRNA.	NM_000848	2	1
**HIST1HID:** histone cluster 1, hld	NM_005320	3	1
**HNRPK:** heterogeneous nuclear ribonucleoprotein K. pre-mRNA processing. Cell cycle progression	NM_031262	2	1
**IDH1**: Homo sapiens isocitrate dehydrogenase 1 (NADP+), soluble, mRNA.	NM_005896	2	1
**KIF4A:** kinesin family member 4. Involved in many crucial cellular processes, including cell division.	NM_012310	3	1
**OR5I1:** olfactor receptor, family 5, subfamily I. Initiate a neuronal response that triggers the perception of smell.	NM_006637	3	1
**OTUD4:** OTU domain containing 4 protein isoform 2.	NM_017493	2	1
**PEX12:** peroxisomal biogenesis factor 12. Required for protein import into peroxisomes.	NM_000286	2	1
**PPP2R1B:** beta isoform of regualtory subunit A, protein phosphatase 2. Implicated in the negative control of cell growth and division.	NM_002716	2	1
**PTPRF/PPFIA1:** The protein encoded by this gene is a member of the LAR protein-tyrosine phosphatase-interacting protein (liprin) family	NM_002840	3	1
**RAB31:** RAB31, member RAS oncogene family.	NM_006868	2	1
**REL:** v-rel reticuloendotheliosis viral oncogene, a transcription factor that is a member of the **Rel**/NFKB family,	NM_002908	2	1
**RFC4:** Homo sapiens replication factor C (activator 1) 4 (37kD), mRNA.	NM_002916	2	1
**SCARB1:** scavenger receptor class B, member 1 isoform 1.	NM_005505	2	1

Gene and Function	EntrezGene ID	#Clones	#Sorts
**SIRT1:** sirtuin 1. Functions in humans unknown, in yeast they regulate epigenetic gene silencing and suppress recombination of rDNA.	NM_012238	3	1
**SLC2A6:** solute carrier family 2 (facilitated glucose transporter), member 6.	NM_017585	2	1
**TNFRSF6B:** tumor necrosis factor receptor superfamily, member 6b. Plays a regulatory role in suppressing FasL- and LIGHT-mediated cell death.	NM_032945	5	1
**UBE2E3:** ubiquitin-conjugating enzyme E2E 3. Involved in targeting abnormal or short-lived proteins for degradation.	NM_006357	3	1
**UPK1A:** uroplakin 1A; Mediate signal transduction events that play a role in the regulation of cell development, activation, growth and motility.	NM_007000	3	1
